# New Products Generated from the Transformations of Ferulic Acid Dilactone

**DOI:** 10.3390/biom10020175

**Published:** 2020-01-23

**Authors:** Ying He, Yuan Jia, Fachuang Lu

**Affiliations:** 1 State Key Laboratory of Pulp and Paper Engineering, South China University of Technology, 381 Wushan Rd., Tianhe District, Guangzhou 510640, China; 201720126333@mail.scut.edu.cn; 2 Guangdong Engineering Research Center for Green Fine Chemicals, Guangzhou 510640, China; 201620121278@mail.scut.edu.cn

**Keywords:** dehydrodimers, alkali/acid treatment, GC-MS, NMR, radical coupling

## Abstract

Various ferulic acid (FA) dimers occurring in plant cell walls, such as 8-5-, 8-O-4-, 5-5-, and 8-8-coupled dimers, are effective antioxidants and potential antimicrobials. It is necessary to access these diferulates as reference compounds to validate those isolated from plants. 3,6-bis(4-hydroxy-3-methoxyphenyl)-tetrahydrofuro-[3,4-c]furan-1,4-dione, a 8-8-coupled FA dilactone generated from ferulic acid via radical coupling, has been used to synthesize 8-8-coupled FA dimers although few reports investigated the distribution of products and mechanisms involved in the transformation of FA dilactone. In this work, the FA dilactone, obtained from FA by a peroxidase-catalyzed radical coupling, was reacted under various base/acid conditions. Effects of reaction conditions and workup procedures on the distribution of products were investigated by GC-MS. The isolated products from such treatments of FA dilactone were characterized by NMR. New derivatives of FA dimer including 2-(4-hydroxy-3-methoxybenzylidene)-3-(hydroxyl-(4-hydroxy-3-methoxyphenyl)methyl)succinic acid and 2-(bis(4-hydroxy-3-methoxyphenyl)-methyl)-succinic acid were produced from NaOH treatment. Another novel 8-8-coupled cyclic FA dimer, diethyl 6-hydroxy-1-(4-hydroxy-3-methoxyphenyl)-7-methoxy-1,2-dihydronaphthalene-2,3-dicarboxylate was identified in products from FA dilactone treated by dry HCl in absolute ethanol. Mechanisms involved in such transformations were proposed.

## 1. Introduction

Natural phenolics, the secondary metabolites extracted from plants, such as gallic acid [1] and ellagic acid [2], have been shown to have various biological activities including antioxidant, antimicrobial, and anticancer capacities [3,4]. Ferulic acid (FA), 4-hydroxy-3-methoxycinnamic acid, one of the large families of biologically active phenolics in plants, is an excellent representative having various applications [5]. In addition to monomeric FA, various FA dimers occurring in plant cell walls, including 5-5-, 8-8-, 8-5-, and 8-O-4-coupled dehydrodimers [6] were demonstrated to have potential antimicrobial properties. FA dimers found in hydrolysate of corn Stover were showed to inhibit the growth of *S. cerevisiae* revealing that poacic acid, an 8-5-coupled decarboxylated ferulic acid dimer (8-5 DC), had the greatest antifungal activity with an IC_50_ of 111 μg/mL (324 μM) against *S. cerevisiae*. This inhibition is comparable to that of the widely used fungicides picoxystrobin (IC_50_ of 308 μM) or polyoxin D (IC_50_ of 340 μM) and substantially lower than that of the primary fungicide used in organic agriculture, copper sulfate (IC_50_ of 2.4 mM) [7]. Some dimeric ferulates showed higher radical-scavenging efficacy than the monomers [8].

The rich structural diversity of 8-8-coupled FA dimers have attracted considerable attention in recent decades because of their unique architectures and diverse biological activities [9,10]. However, extracting and isolating such dehydrodimers on a large scale from plants is challenging due to their low abundance [11]. It is desirable and needed to devise synthetic strategies to make FA dimers with potential antimicrobial capabilities. 3,6-bis(4-hydroxy-3-methoxyphenyl)-tetrahydrofuro-[3,4-c]furan-1,4-dione, a FA dilactone, was first synthesized by Cartwright [12] using FeCl_3_. It also can be conveniently prepared from an enzymatic oxidative coupling of ferulic acid [12,13].

**Figure 1:**
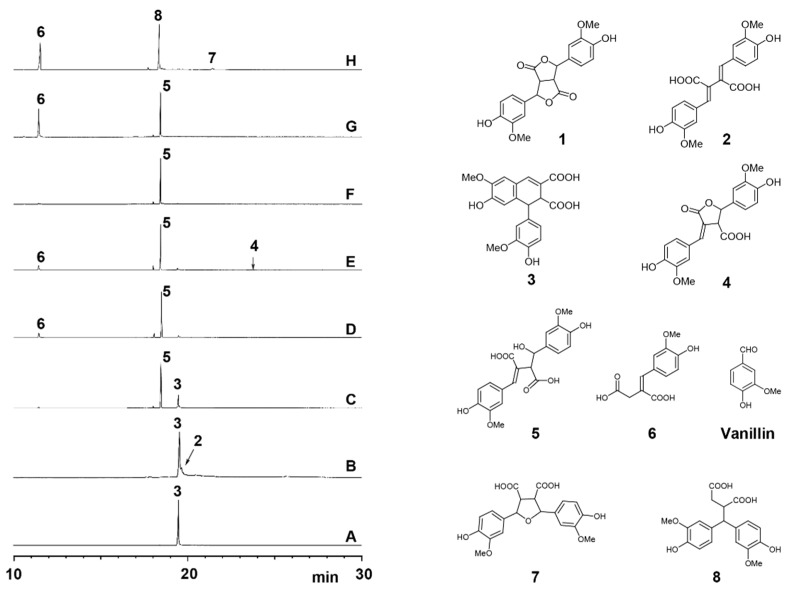
Left: Total ion chromatograms of crude products obtained from transformation of FA dilactone by 12-h alkali treatments: (**A**) 4 eq. 0.1M NaOH; (**B**) 15 eq. 0.1M NaOH; (**C**) 15 eq. 0.5 M NaOH; and (**D**–**H**) 120 eq. NaOH (**D**) 1.0 M; (**E**) 2.0 M; (**F**) 4.0 M; (**G**) 8.0 M; and (**H**) 16.0 M). Right: The structures of compounds **1**–**8** described in this work.

FA dilactone has been used to prepare 4,8-bis(4-hydroxy-3-methoxyphenyl)-3,7-dioxabicyclo[3.3.0]octan-2-one, a 8-β-coupled dimer formed by radical coupling of ferulic acid or its ester and coniferyl alcohol [14]. Alkali or acid treatment of FA dilactone produced 8-8-coupled diferulic acids (DFA), 8-8-o DFA (compound **2**) and/or 8-8-c DFA (compound **3**) (Figure 1). The FA dilactone has been treated with 2 M NaOH to afford trans-lactone acid **4** as amorphous solid in very high yield following a silica column separation [15]. Acid-catalyzed transformation of the 5-substituted ferulate or their corresponding dilactones produced tetrahydrofuran or dibenzylidenesuccinate types of products [16]. The trans- and cis-isomers of 1,2-dihydronaphthylene diethyl esters can be produced in such a treatment [17]. Acid-catalyzed transformation of FA dilactone **1** in dry methanol was reported to produce trans-1,2-dihydronaphthylene diethyl esters (8-8-c dimethyl diferulic acid ester) [18]. However, no detailed product distributions from such transformations of FA dilactone were reported.

In this work, FA dilactone was reacted under various conditions. Effects of base/acid types, reagent concentrations, and reaction media on the distribution of the resultant products from FA dilactone were investigated by GC-MS and NMR analysis. Products derived from FA dilactone under various treatment conditions were isolated by using Thin Layer Chromatography (TLC) and flash chromatography and were characterized by NMR. Reported here were several new compounds obtained from FA lactone treated under various conditions. Hypothetic mechanisms or pathways leading to those new products were proposed.

## 2. Experimental Section

### 2.1. Materials and Methods

Ferulic acid was a commercial product from Wuhan Yuan-Cheng Gong-Chuang Technology, Co. Ltd. (Wuhan, China). The FA dilactone dimer was synthesized according to an enzyme-catalyzed process (details are included in the Supplemental Materials). NH_4_OH (25%–28% wt), absolute ethanol, 1,4-dioxane, and acetyl chloride were analytical reagent (AR) grade and obtained from Macklin (Shanghai, China). NaOH and Na_2_CO_3_ were also purchased from Macklin (Shanghai, China).

The crude products from preparative-scale experiments using various treatment of FA dilactone were separated by flash chromatography (Isolera One, Biotage, Shanghai) with Snap silica columns or TLC (Silica gel 60 F254, 1 mm plate). The isolated compounds were characterized by NMR (Bruker 600 MHz, in acetone-*d*_6_). NMR spectrum assignments were made according to published data for known compounds or by 2D NMR experiments including COSY, HSQC, and HMBC for new compounds. The NMR data are included in the Supplementary Materials.

High-resolution mass spectra were acquired on a high-resolution mass spectrometer (HRMS, Agilent 1290/maxis impact, Karlsruhe, Germany) with Electron Spray Ionization (ESI) resource. Trimethylsilyl (TMS-) derivatives of products were analyzed by GC-MS (GCMS-TQ8040, Schimadzu, Shanghai). He (1mL·min^−1^) was used as the carrier gas. GC conditions were as follows: SH-Rxi-5Sil MS column (30 m × 25 mm × 25 µm); initial temperature at 200 °C and held for 1 min, and ramped at 10 °C min^−1^ to 280 °C and held for 30 min; injector temperature 250 °C; ionization energy 200 eV; and scan range: m/z 50–700.

### 2.2. Transformations of FA Dilactone under Various Conditions

#### 2.2.1. Alkali Treatments

The FA dilactone (20–60 mg, 0.05–0.15 mmol) was added separately in aqueous base solutions containing given equivalents of bases (Na_2_CO_3_, NH_4_OH, and NaOH) in various concentrations. The reactions were terminated by adding 1 M HCl to acidify the reaction media to pH value around 3. A given amounts of internal standard dissolved in ethyl acetate was added, and the products were recovered by ethyl acetate (EtOAc) extraction. The organic phase was dried over anhydrous MgSO_4_. About 0.2 mL EtOAc containing 3 mg of the crude product were taken for TMS-derivatization and subjected to GC-MS analysis.

For another set of NaOH treatments, the recovered products in ethyl acetate solution (wet, without drying) were evaporated to generate an oil-like product. The products were kept at 25 °C overnight prior to GC-MS analysis (their total ion chromatograms showed significant amounts of product **4**; Figure S1 in Supplementary Materials).

#### 2.2.2. Acid Treatments

**In aqueous solution:** The FA dilactone (50 mg, 0.13 mmol) was dissolved in aqueous dioxane (50% or 99%, v/v) solutions containing HCl in various concentrations. The mixture was stirred at 70 °C for 12 h and was monitored by TLC (n-hexanes/EtOAc, 75:25 v/v). The reaction mixtures were evaporated under reduced pressure to remove all water or solvents. The residues were dissolved in 10 mL absolute ethanol, from which a small amount (0.1 mL) of products was allocated for GC-MS analysis. The ethanol solution was evaporated under reduced pressure to dryness. The residues were redissolved in 10 mL absolute ethanol and esterified by slowly adding 0.5 mL acetyl chloride. The mixture was kept at 25 °C for 24 h. The resultant solution was evaporated to dryness under reduced pressure. The residue was dissolved in acetone and evaporated again (this operation was repeated 2 times). The products were analyzed by NMR.

**In absolute ethanol:** 0.75 mL acetyl chloride was added dropwise into 10 mL absolute ethanol containing 50 mg FA dilactone (0.13 mmol). The reaction mixture was stirred at 25 °C for 12 h when the resultant yellow solution was evaporated under reduced pressure to dryness. The residues were dissolved in 20 mL EtOAc and washed with saturated NH_4_Cl. The EtOAc solution was dried over anhydrous MgSO_4_ and filtered off to remove the solids. The products were obtained after evaporating off the EtOAc solvent and were analyzed by NMR.

## 3. Results and Discussion

### 3.1. Transformation of Dilactone in Alkali Solutions

**Figure 2:**
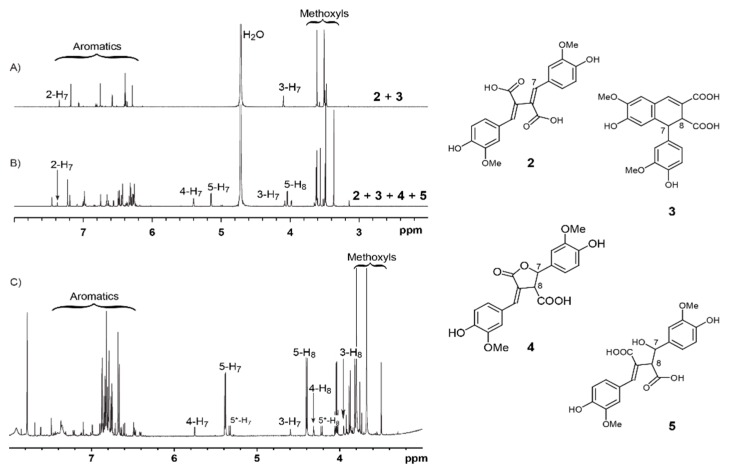
In situ ^1^H NMR (in D_2_O) of products from ferulic acid (FA) dilactone treated by (**A**) 1 M Na_2_CO_3_ and (**B**) 1 M NaOH. (**C**) ^1^H NMR (in Acetone-*d*_6_) of crude products recovered from 2 M NaOH treatment of FA dilactone.

It was reported that lactone product **4** (Figure 1) was obtained in high yield by a 2 M NaOH treatment of FA dilactone at 25 °C followed by a silica column separation [15]. FA dilactone was reacted with 1 M or 2 M aqueous Na_2_CO_3_ at 25 °C overnight. producing compounds **2** and **3** completely (Figure S2). We were wondering why the lactone **4** seems pretty stable in 2 M NaOH solution at 25 °C whereas, in aqueous 1 M Na_2_CO_3_, no lactone product **4** was obtained from the FA dilactone. To solve this puzzle, the 2 M NaOH treatment of FA lactone was revisited. The crude products from such a treatment (with 2 M NaOH for 16 h) were recovered by ethyl acetate solvent extraction after acidification and directly analyzed by GC-MS. It was surprising that the major product was compound **5** along with small amounts of compound **6** and trace amounts of **3** (Figure 1). NMR analysis of the crude products also confirmed the GC-MS result (Figure 2). The NMR analysis suggested that compound **5** is a hydration product of compound **2**. The mass spectrum of TMS-derived **5** has a base ion fragment (m/z) of 297, suggesting that a benzylic hydroxyl (C_7_-OH) group is present in this compound. Its high-resolution mass data also supported the formula given in the suggested structure of **5**.

**Figure 3:**
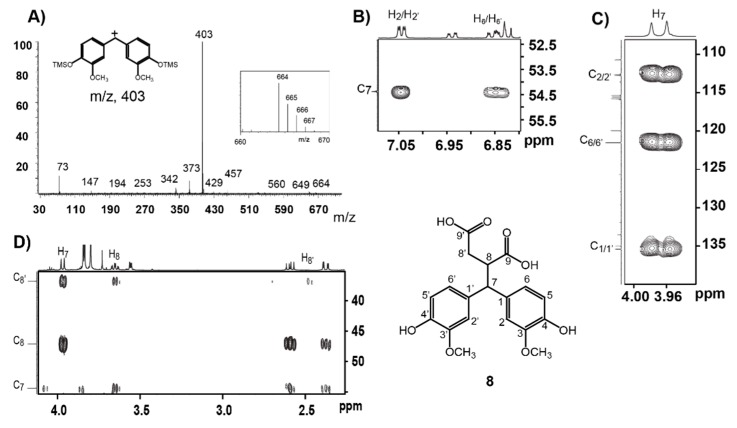
HMBC NMR (**B**–**D**) and mass spectra (**A**) of compound **8**, showing characteristic features to establish its chemical structure shown.

More surprising was that almost no lactone product **4** was detected (Figure 1 left panel, E) although NMR of the crude products proved the presence of compound **4** (Figure 2). This interesting finding promoted further study on transformation of the FA dilactone under various alkali conditions. Thus, effects of NaOH concentrations and its stoichiometry on the transformations of FA dilactone were investigated. The total crude products recovered by ethyl acetate solvent extraction after acidification were directly silylated and analyzed by GC-MS (Figure 1). In 0.1 M NaOH solution, using less than 15 eq. of NaOH, the major product was compound **3**, 8-8-c diferulic acid, whereas compound **5**, a hydrolysis product, was the main product when in 0.5 M NaOH using 15 eq. of NaOH. When large excess amounts of NaOH (120 eq.) were used, the dominate product was still compound **5** even though the NaOH concentrations used varied from 1 M to 4 M. Higher concentrations resulted in large amount of compound **6**, a retro-claisen condensation product. Methyl ether of **6**, veratrylidenesuccinic acid, has been obtained from NaOH treatment of dimethyl ether of dilactone **1** before [12]. However, at the concentration (16 M) close to saturation point, a new major product, compound **8**, was generated from FA dilactone **1** although compounds **6** and **7** were also detected. In total, seven products, compounds **2**–**8**, were isolated by silica column separation from preparative scale experiments and characterized by NMR (see Supplementary Materials for detailed NMR data). Compounds **2** and **3** were isolated from 0.1 M NaOH treatment. Compound **4** was hardly detected by GC-MS in products generated from any NaOH treatment of dilactone **1** (Figure 1). This contrasted the previous report that compound **4** was obtained in high yield from a 2 M NaOH treatment [15]. However, in an experiment where oil-like products obtained by evaporating the wet (no drying) ethyl acetate solution of products were kept at 25 °C overnight, compound **4** was found to be one of the major products while **5** decreased significantly, as shown by GC-MS analysis (Figure S1, Supplementary Material). This suggested that compound **5** was not stable and converted to compound **4** and/or compound **6** under the storage conditions. It was also found that compound **4** can be formed, apparently from compound **5**, during column separation or by mixing with silica gel in ethyl acetate (Figure S3). All these results above suggest that the majority of compound **4** reported might not be formed directly from the NaOH treatment; instead, it was actually generated during posttreatments including silica column separation. In situ NMR experiment also indicated that major species derived from FA dilactone **1** in 1 M NaOH D_2_O solution (excess amount) is compound **5** while the rest of the minor ones are compounds **2**–**4** (Figure 2B). Compound **7**, a ferulic acid dimer with a symmetry tetrahydrofuran (THF) ring, was identified here for the first time from 16 M NaOH treatment of **1**. It was verified by comparing its GC retention time, mass spectrum, and NMR data with those of the reference compound synthesized according to a published method [19]. Compound **8**, a rearrangement product, is also a new compound derived from FA dilactone **1**. The TMS-derivative of compound **8** (Figure 3A) has a molecular ion (m/z) of 664, which is not typical of any 8-8-coupled FA dimers reported before. The base ion fragment (m/z) of compound **8** (TMS-derived) was 403, implying that product **8** has a bis-guaiacyl methylene structure. Although being mixed with minor compound **7**, it was successful to figure out the structure of compound **8** by 2D NMR experiments (Figure 3). From the HMBC spectrum of compound **8** (Figure 3B), it can be seen that the C_7_ signal (54.38 ppm) correlates to two sets of aromatic proton signals (6.85 ppm and 7.05 ppm, assigned to protons at C_6_ and C_2_ of guaiacyl units. In addition, what can be seen (Figure 3C) is that ^1^H signal at 3.97 ppm (assigned to H_7_) correlates to three pairs of ^13^C signals (134.98/135.40 ppm, 112.60/112.75 ppm, and 121.51/121.51 ppm) corresponding to C_1_, C_2_, and C_6_ of guaiacyl units. Further examination of the NMR (Figure 3D) suggested that ^1^H signals at 3.97 ppm, 3.65 ppm, and 2.59–2.38 ppm are correlated with each other and can be assigned to aliphatic protons at C_7_, C_8_, and C_8′_, respectively. High-resolution mass spectrum also supported the formula of the structure of compound **8** as shown in Figure 3.

**Figure 4:**
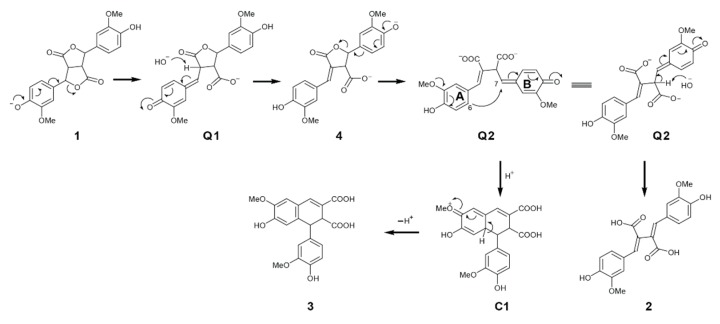
The proposed mechanisms leading to the formation of compounds **2** and **3**.

All these products were quantified by GC-TIC with their GC-MS response factors against an internal standard, (E)-4-(4-(4-hydroxy-3-methoxyphenyl)but-3-en-2-yl)-2-methoxyphenol (vinlyguaiacol dimer) (Table 1). Na_2_CO_3_ and NH_4_OH with various concentrations were also used for treatment of dilactone **1**, and their results are listed in Table 1. Apparently, the ratios of the products from FA dilactone **1** treated by various concentrations (1 M to 5 M) of Na_2_CO_3_ at 25 °C did not change that much. This could be explained by the fact that the basicity of aqueous Na_2_CO_3_ does not change much in concentrations ranging from 1 M to 5 M due to its buffer capacity (pHs of 1 M, 2 M, and 5 M Na_2_CO_3_ solutions are 11.38, 11.55, and 11.72, respectively). However, ammonia concentrations used for treating dilactone **1** produced various ratios of the products **2** and **3**, showing that higher ammonia concentration favored the formation of **2**. This is because higher concentrations of ammonia (hydroxide ion) would favor the elimination of proton H_7_ from **Q2** (Figure 4), leading to the formation of compound **2**.

**Figure 5:**
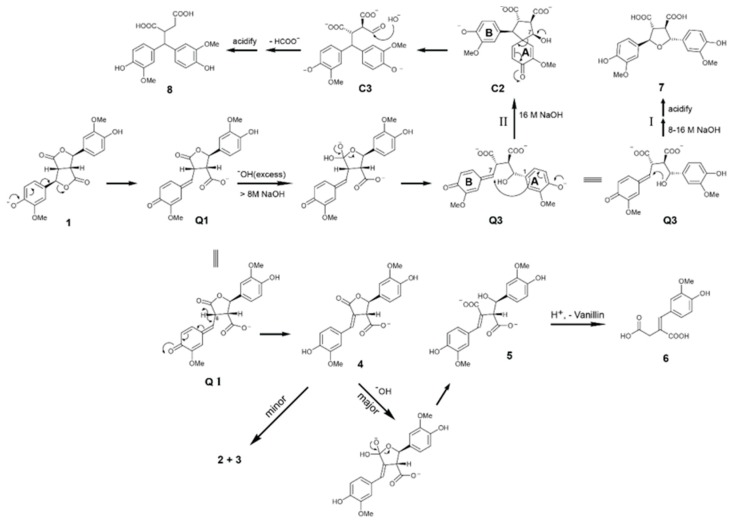
The proposed mechanisms leading to the formation of products from FA dilactone in aqueous NaOH solution followed by acidification. Note: all compounds are racemic although one isomer is drawn.

As for how these products were formed from FA dilactone under aqueous alkali treatment conditions, a plausible mechanism or pathways were proposed and illustrated in Figure 4 and Figure 5. In Na_2_CO_3_, NH_4_OH, or diluted NaOH solution, the FA dilactone was converted into quinomethide **Q1** that loses a proton from 8-position readily to produce lactone **4**, from which quinomethide **Q2** was produced through opening the lactone by electron pushing. Two possible options for stabling **Q2**. One is a nucleophilic attack from carbon-A_6_ to the 7-position of B-ring to form compound **3** eventually through aromatization of intermediate **C1**. The other is an elimination of a proton from the 8-position producing compound **2** (Figure 4).

As Figure 5 shows, in excess NaOH solutions with molar concentrations beyond 0.5 M, dilactone **1** was prone to quickly forming quinone methide **Q1**. As an intermediate produced from **Q1** by elimination of one H_8_, compound **4** was hydrolyzed quickly, giving rise to a major product, compound **5**, which is suggested or supported by the fact that one dominate diastereomer was detected by GC-MS (Figure 1) or by in situ NMR (Figure 2). In higher (8 M and beyond) concentrations of NaOH, however, direct hydrolysis of **Q1** is a fast and dominate reaction generating **Q3** (Figure 5). From **Q3**, two possible pathways lead to the formation of products **7** or **8**. In one direction, the newly formed hydroxyl group attacks the C_7_ of B-ring forming product **7** (pathway I). In another direction (pathway II), the C_1_ of A-ring competitively attacks the 7-position of the B ring driven by electron pushing from phenoxide of A-ring, forming a spiro-dienone intermediate B, which was then rearranged through cleavage of C_1_–C_7_ bonds by the electron pushing from 7-OH of A-ring. The resultant aldehyde **C3** is attacked by a hydroxide ion, eliminating a formate to produce compound **8** eventually (Figure 5). Meanwhile, vanillin and compound **6** were also produced from compound **5** through retro-claisen condensation during acidification.

### 3.2. Transformation of Dilactone in Acidic Solutions

**Figure 6:**
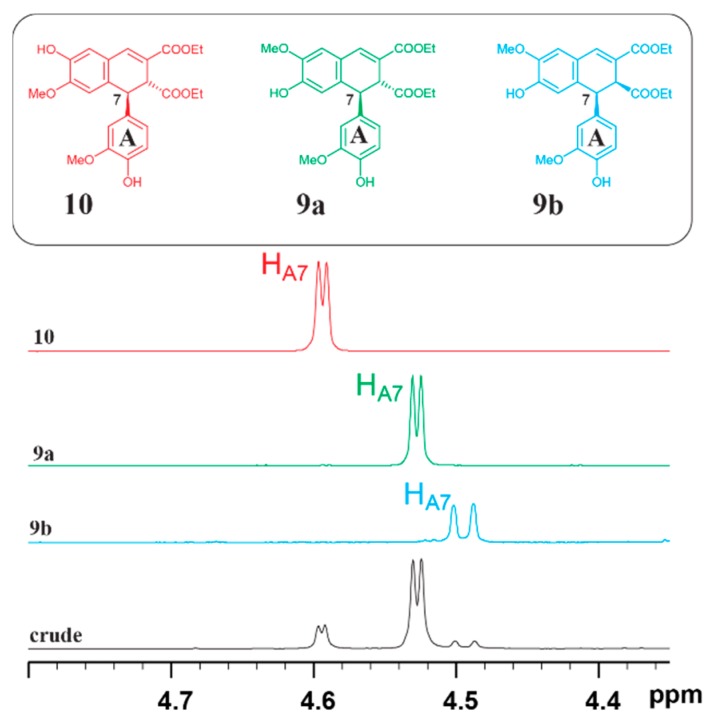
Partial ^1^H NMR spectra showing the characteristic chemical shifts of A-7 protons in crude products from acid catalyzed transformation of FA dilactone and compounds **9** and **10**.

Compound **3** (8-8-c diferulic acid) belongs to an important group of arylnaphthalene lignans with diverse architectures and biological activities [20,21]. Acid-catalyzed transformation of FA dilactone **1** in absolute alcohol (methanol) produced 8-8-c diferulic acid ester, as reported by Takei et al [18]. However, no other products have ever been reported from such acid catalyzed transformations. In the current study, FA dilactone **1** was treated with dry HCl (generated from acetyl chloride and absolute ethanol) in ethanol at 70 °C for 12 h; the crude products were characterized by GC-MS and NMR after completely removing all volatile chemicals. Although GC-MS chromatogram of the crude products showed one major peak accompanied with a minor one (Figure S4, Supplementary Materials), NMR spectrum of the crude products indicated that there was an unknown compound of which the ^1^H chemical shift at around 4.6 ppm, tentatively assigned to A_7_-proton, was different from those of the well-documented trans- and cis-isomers of 8-8-c diferulic acid ethyl esters **9a** and **9b** (Figure 6). Our curiosity led to an effort to reveal the identity of the unknown product. Thus, the crude product of a preparative scale reaction was subjected to a flash silica column separation with n-hexanes/EtOAc (50:50, v/v) as eluent resulting in two fractions. The major fraction contained compound **9**, from which the two isomers (**9a** and **9b**) were separated by TLC. The minor fraction was identified by 2D NMR analysis to be compound **10** of which the structure is shown in Figure 6.

The detailed NMR assignment of compound **10** can be found in the Supplementary Materials (Figures S5 and S6). To the best of our knowledge, it was the first time that compound **10** was discovered in products obtained by such a treatment.

**Figure 7:**
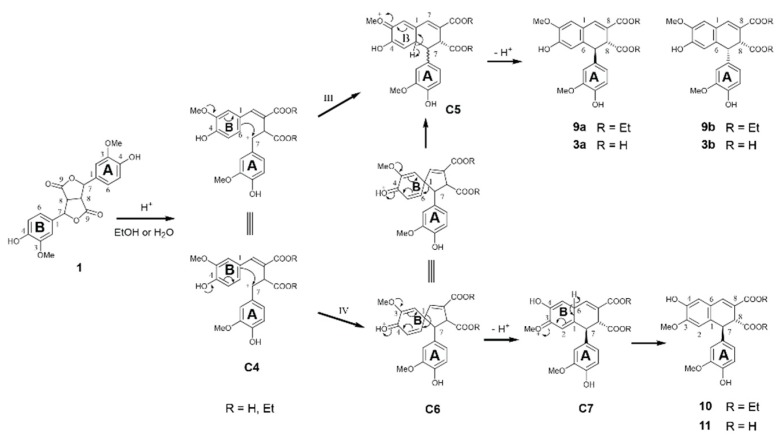
Proposed mechanisms involved in acid treatment of FA dilactone leading to the formation of compounds **10** and **11**.

Figure 7 shows the plausible pathway leading to the formation of compounds **9** and **10** from FA dilactone **1**. In acidic solutions, the intermediate cation **C4** is formed from FA dilactone **1**. Nucleophilic attacking from B-ring at its 6-position to the 7-position of A-ring forms cation **C5** (pathway III). The elimination of a proton from **C5** leads to the formation of compound **9** (**9a** and **9b**). Meanwhile, a competing reaction (pathway IV) is the nucleophilic attacking from B-ring at its 1-position to the 7-position of A-ring, driven by electron pushing from phenolic hydroxyl on B-ring, generating a spiro-dienone **C6**. Electron pushing from the methoxyl group on the B-ring of **C3** would rearrange bonding on the 1-position of the B-ring so that two cations (**C5** and **C7**) are possibly formed, as shown in Figure 7. Re-aromatization of **C7** produces compound **10**. The molar ratios of these three isomeric products generated in dry 1 M HCl absolute ethanol of FA dilactone was determined by ^1^H NMR to be 73.4% (**9a**):8.2% (**9b**):18.3% (**10**).

In acidic (0.5 M HCl) aqueous 1,4-dioxane (50%) solution, dilactone **1** was dominantly transformed to compound **3** although there were small amounts of other products which were difficult to identify by either GC-MS or NMR. Therefore, the crude product was esterified by dry HCl in absolute ethanol and characterized by ^1^H NMR. Comparing the NMR spectrum of the esterified crude product to that of compounds **9** and **10** confirmed that three compounds, **3a**, **3b**, and **11**, were indeed produced (Figure S7, Supplementary Materials). The molar ratios of these dimers were determined by integrating their characteristic signals (assigned to H_7_ of A-ring) at around 4.5–4.6 ppm and are listed in Table 2.

When acid concentration in reaction media was increased from 0.5 to 1.2 M, the percentage of dimer **11** was increased from 4.1% to 22.7%, suggesting that higher acid concentration favors the formation of compound **11**. At the same acid concentration (1.2 M), decreasing water content of the reaction media produced more compound **11**. The reaction mechanisms involved for the transformation of FA dilactone in aqueous acid treatment can also be described similarly in Figure 7.

In summary, the identified new compounds in the current study implicate that the easily available FA dilactone **1** poses as an excellent starting material to access diverse diferulic acids. More importantly, compounds produced in the transformation could be considered as “plant-derived” chemicals potentially with good biological activities (studies in this regard are undergoing promising progress), such as antibacterial, antioxidant, and anti-inflammatory abilities.

## 4. Conclusions

In this study, FA dilactone was reacted under various alkali/acidic conditions to produce degradation products or diferulates with diverse structures. Chemicals (bases or acid), their stoichiometry and concentrations, and reaction media were factors influencing the transformation outcome and reaction pathways leading to various products. New derivatives including 2-(4-hydroxy-3-methoxybenzylidene)-3-(hydroxy(4-hydroxy-3-methoxyphenyl)methyl)succinic acid (compound **5**), (trans, trans, trans)-2,5-bis(4-hydroxy-3-methoxyphenyl)-tetrahydrofuran-3,4-dicarboxylic acid (compound **7**), 2-(bis(4-hydroxy-3-methoxyphenyl)methyl)succinic acid (compound **8**), and diethyl 6-hydroxy-1-(4-hydroxy-3-methoxyphenyl)-7-methoxy-1,2-dihydronaphthalene-2,3-dicarboxylate (compound **10**) from FA dilactone were discovered and identified. Reaction pathways involving spiro-dienone intermediates were proposed to be responsible for the formation of the newly discovered products from the transformation of FA dilactone under various alkali/acidic conditions.

## Figures and Tables

**Table 1:** The products and yields (%) obtained under various base concentrations.

Base Concentrations	Eq.	Temp. (°C)	Time (h)	Products (%)					
				2	3	5	6	7	8
0.1 M NaOH	3	25	16	5.8	91.4				
0.1 M NaOH	4			5.6	92.1				
0.1 M NaOH	15			6.0	89.3				
0.5 M NaOH	15			2.8	65.4	21.2	4.0		
1 M NaOH	120			3.0	7.6	79.5	3.3		
2 M NaOH				2.4	8.7	78.9	3.7		
4 M NaOH						87.2	0.8		
8 M NaOH						62.5	23.4		3.7
16 M NaOH							21.8	15.5	60.2
1 M Na_2_CO_3_	120	25	16	8.7	88.7				
2 M Na_2_CO_3_				9.2	89.5				
5 M Na_2_CO_3_				8.7	89.2				
0.5 M NH_4_·OH	120	25	16	31.5	67.7				
5 M NH_4_·OH				42.4	56.8				
14 M NH_4_·OH				50.9	46.5				

**Table 2:** The molar ratios of products from FA dilactone under various acidic conditions *.

Acid Concentrations	Reaction Media	Ratios of Products (%)
1.0 M HCl	Dry HCl in ethanol	**10** (18.3):**9a** (73.4):**9b** (8.2)
0.5 M HCl	50% dioxane	**11** (4.1):**3a** (89.2):**3b** (6.6)
1.2 M HCl	50% dioxane	**11** (22.7):**3a** (70.5):**3b** (6.8)
1.2 M HCl	99% dioxane	**11** (27.8):**3a** (63.8):**3b** (8.3)

* Note: reactions were performed at 70 °C for 12 h. The ratios were determined by ^1^H NMR. The ratios among **11**, **3a**, and **3b** were determined according to the corresponding ratios among **10**, **9a**, and **9b** after esterification.

## Supplementary Materials

The supplementary materials are available online at https://www.mdpi.com/2218-273X/10/2/175/s1.
