# Supplementary material for: New Products Generated from the Transformations of Ferulic Acid Dilactone
Source: Biomolecules. 2020 Jan 23;10(2):175. doi: 10.3390/biom10020175 (PMC7072328; doi:10.3390/biom10020175)
Supplement: Supplementary file 1 [file biomolecules-10-00175-s001.pdf]

Supporting Information For

New Products Generated from the Transformations of Ferulic Acid Dilactone

Synthesis of the Dimer β-β Dilactone

The ferulic acid (200 g, 1.03 mol) was dissolved in solution (6 L ethanol and 24 L H_2O), to which subsequently 200 mL of 30% H_2O_2 (64 g, 0.567 mol, 0.55 eq.) and horseradish peroxidase (150 mg) were added. After stirring for 2 h in room temperature, the precipitated solid was filtrated off. The air-dried crude product was purified by crystallization in ethyl acetate (EtOAc). A pale yellow solid product (51 g, 25% yield) was obtained.

Quantitation of Alkali Treatment Products by GC-MS with an Internal Standard

The internal standard (IS) used was vinylguaiacol dimer (its structure is shown below).

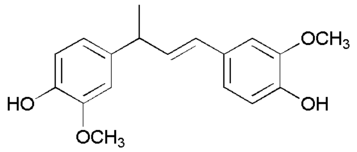

Trimethylsilyl (TMS) derivatization: The given amount of IS and purified reference dimers were dissolved into 800 μL ethyl acetate in a GC vial; then, pyridine (100 μL) and N,O-bis trimethylsilyl trifluoroacetamide (BSTFA, 98%, 100 μL) were added. Then, the mixtures were trimethylsilyl derivatized at 55 °C and kept for 40 min[1]. Finally, the TMS derivatives were subjected to GC-MS analysis.

The response factor was calculated by the peak area by using the following equation:

$$f = \frac{\frac{A_s}{m_s}}{\frac{A_r}{m_r}}$$

where A_s is the peak area of the internal standard substance, A_r is the peak area of dimers, m_s is the mass of the internal standard substance, and m_r is the mass of dimers.

At the end of the reaction, the internal standard substance was added to the mixture. After extraction, other treatments, and TMS derivatization, the mixture was subjected to GC-MS. The mass of dimers can be calculated by the following equation:

$$m_i = f \times \frac{A_i}{\frac{A_s}{m_s}}$$

where m_i is the mass of the internal standard and A_i is the peak area of the dimer to be measured.

Table 1. The calculated response factors of individual compounds against the internal standard (IS).

Dimer	1	2	3	4	5	6	7	8
response factor	7.1967	3.7962	7.1967	12.6240	8.4525	1.2925	3.0388	1.8646

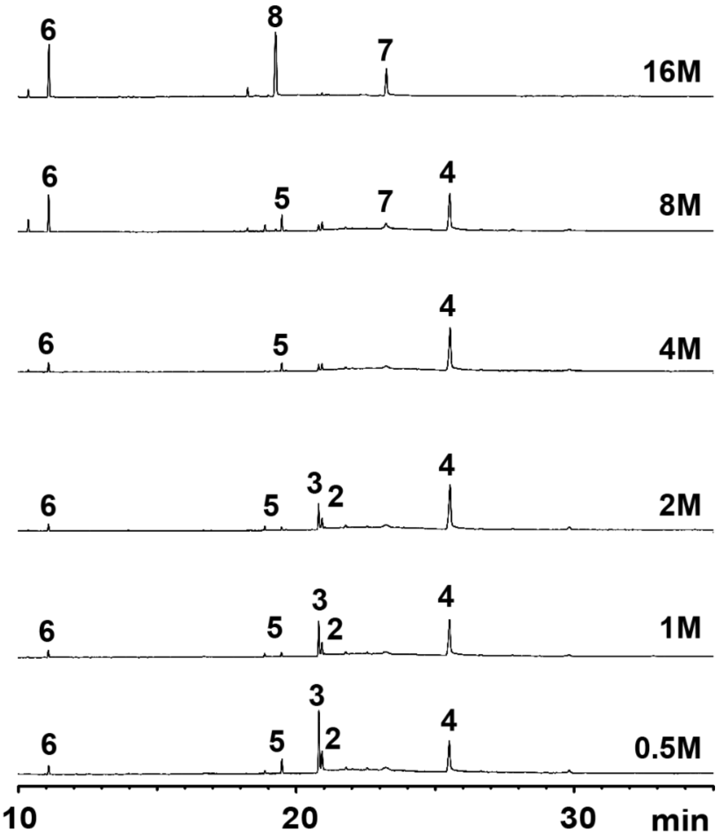

Figure S1. Total ion chromatography of products from NaOH treatment with various concentrations. Workup procedure: After neutralization, the products were recovered by ethyl acetate (EtOAc) extraction. Oil-like products were obtained by evaporating the wet (no drying) ethyl acetate solution of products was kept at 25 °C overnight; compound **4** was found to be one of the major products while **5** almost disappeared.

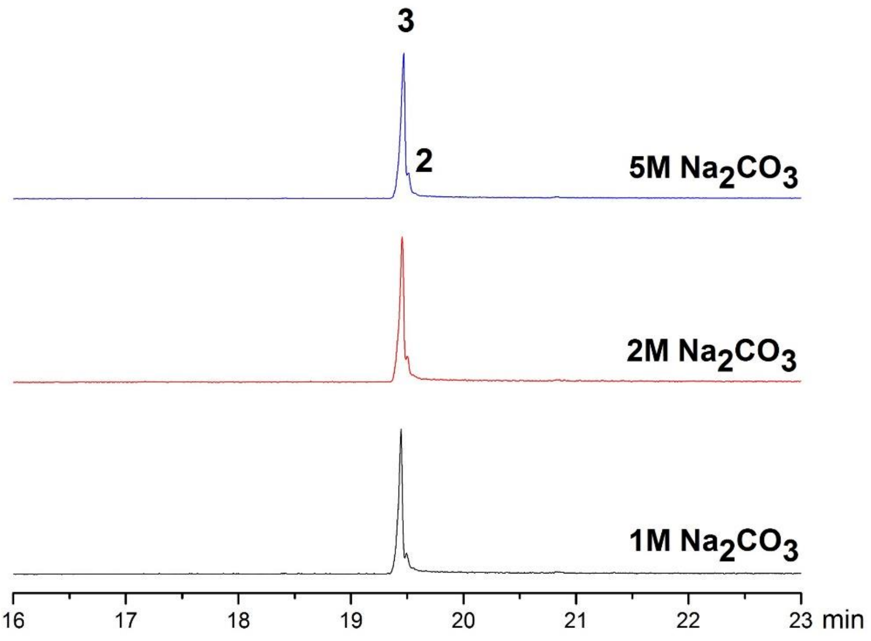

Figure S2. Total ion chromatograms of crude products from ferulic acid (FA) dilactone treated by 1 M, 2 M, and 5 M aqueous Na_2CO_3 at 25 °C overnight, showing that the ratios between products **2** and **3** did not change with Na_2CO_3 concentrations.

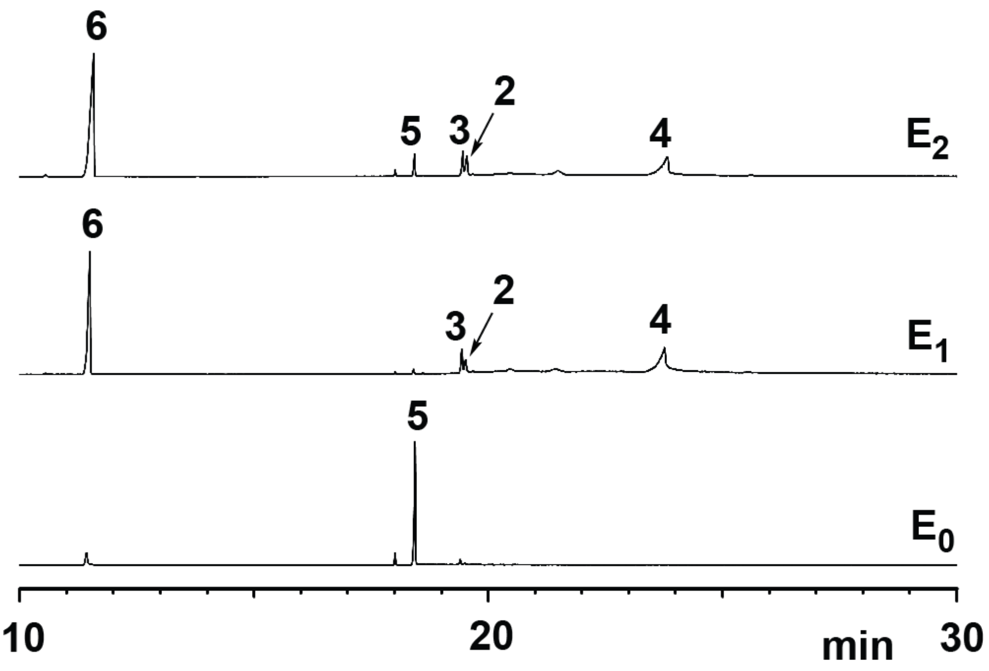

Figure S3. Total ion chromatograms of crude products from FA dilactone treated by 2 M NaOH solution. E_0: products recovered by ethyl acetate (EtOAc) extraction and dried over anhydrous $MgSO_4$; E_1: products in ethyl acetate (EtOAc) were mixed with silica gel and stirred overnight; and E_2: products of E_0 were loaded in a silica column and slowly (90 min) eluted with ethyl acetate (EtOAc) containing 1% acetic acid (HOAc).

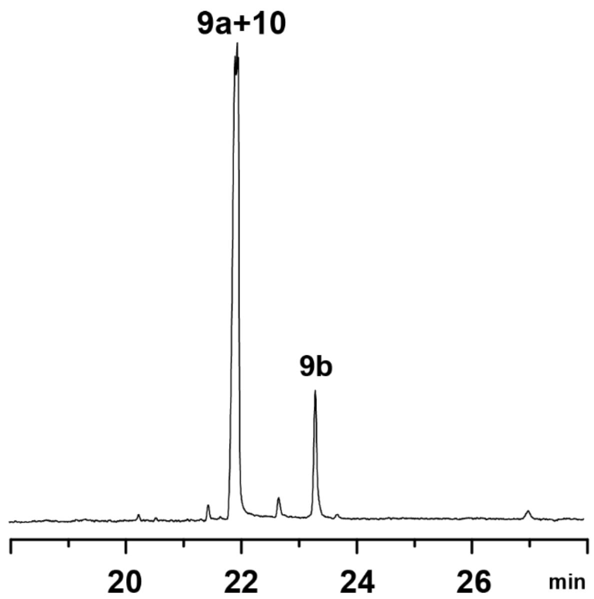

Figure S4. Total ion chromatogram of crude products from FA dilactone treated by dry HCl in absolute ethanol, showing major three products: **9a**, **9b**, and **10.**

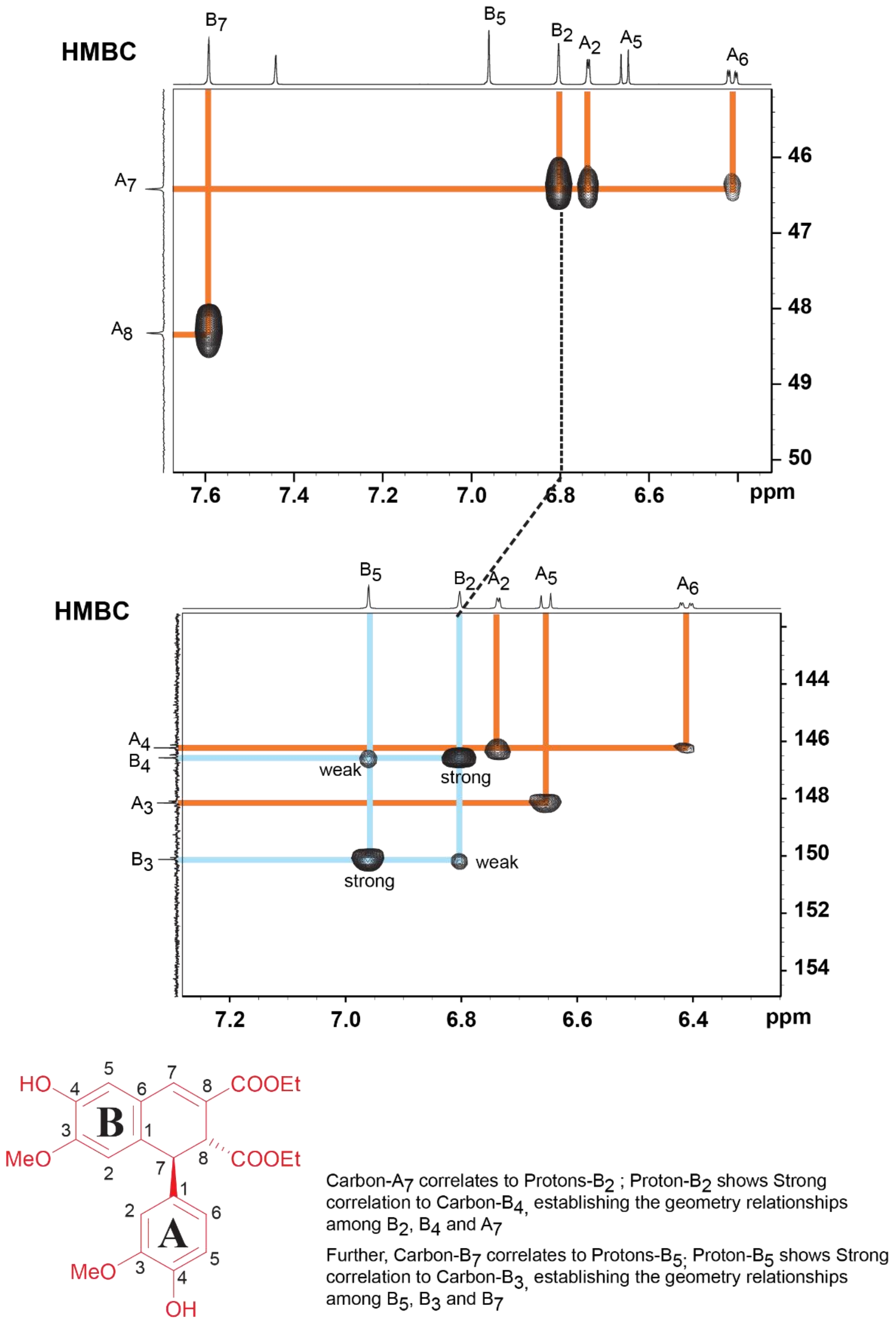

Figure S5. Partial HMBC spectrum of compound **10**, showing the characteristic correlations between carbons and protons of B-ring.

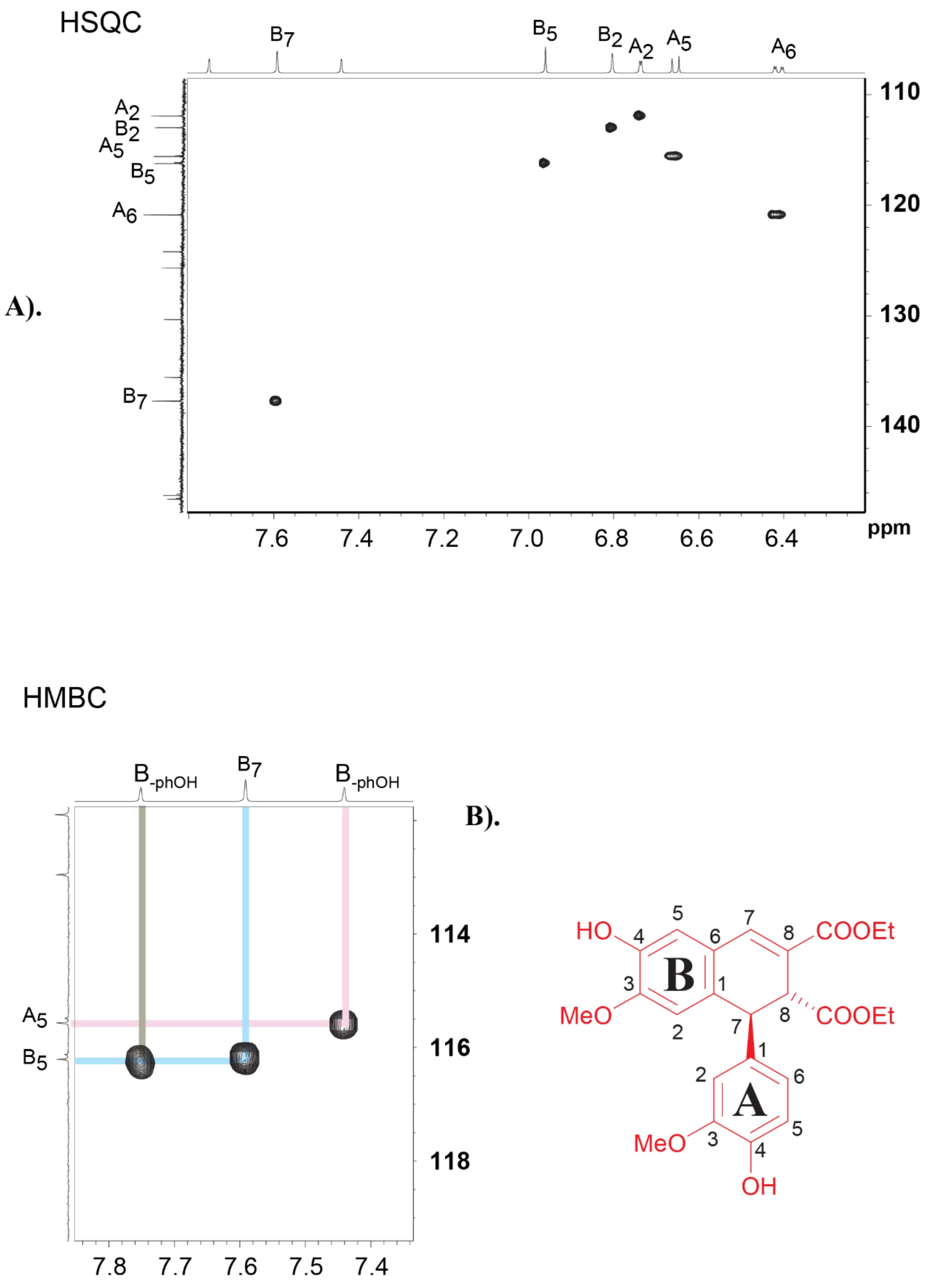

Figure S6. The partial HMBC NMR showing the correlation between carbon B_5 and proton B_7 as well as phenolic OH of B-ring to establish spatial their relationships: HSQC spectrum (top) shows all C–H correlations of protonated carbons in compound **10**.

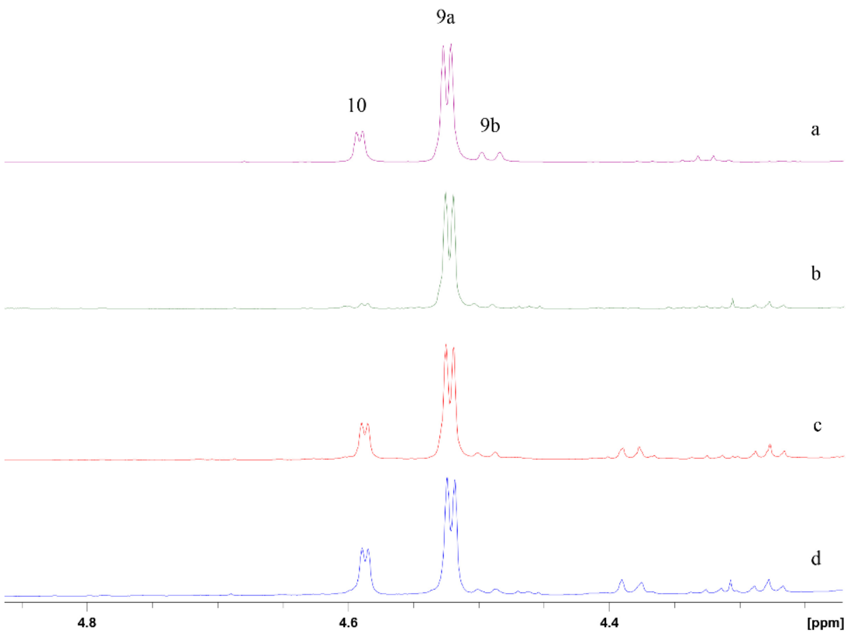

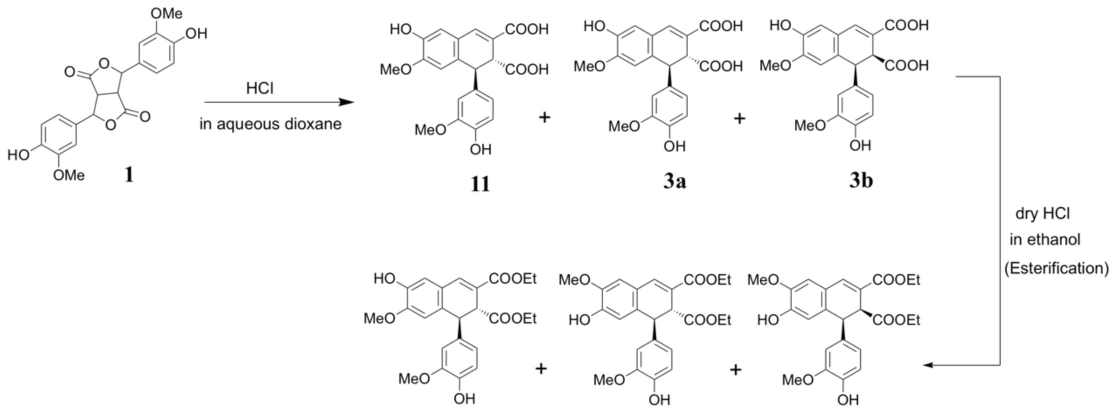

Figure S7. Partial ^{1}H NMR of esterified products from aqueous HCl treatment of FA dilactone, showing the characteristic chemical shift of proton A_7 of compounds **9a**, **9b**, and **10**, which were formed from **3a**, **3b**, and **11** correspondingly by esterification. (**a**) Products from 1.0 M HCl (dry) in absolute ethanol; (**b**) products from 0.5 M aqueous HCl (10 mL 50% dioxane) treatment; (**c**) products from 12 M HCl (10 mL 50% dioxane) treatment; and (**d**) products from 12 M HCl (10 mL 99% dioxane) treatment.

Product Characterization

Compound **1–4** and **9a** have been reported in the literature [2, 3].

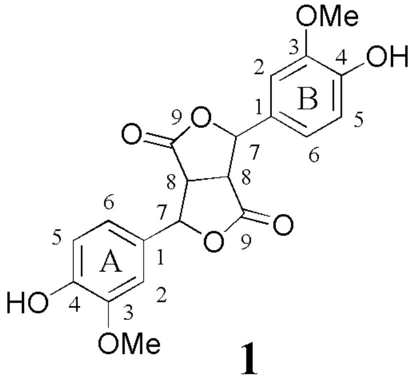

1

Table 2. ^{1}H and ^{13}C NMR data of compound **1** (Acetone d$_6$, 600 MHz for ^{1}H NMR and ^{13}C NMR, chemical shift δ in ppm, coupling constant *J* in Hz).

Position	δH (ppm)	δC (ppm)
3-OMe	3.86, s	56.3
1		130.83
2	7.05 (d, *J* = 2.0 Hz)	110.31
3		148.20
4		148.73
5	6.86 (d, *J* = 8.0 Hz)	116.01
6	6.92 (dd, *J* = 8.0, 2.0 Hz)	119.58
7	4.07 s	83.21
8	5.77 s	49.08
9		175.0

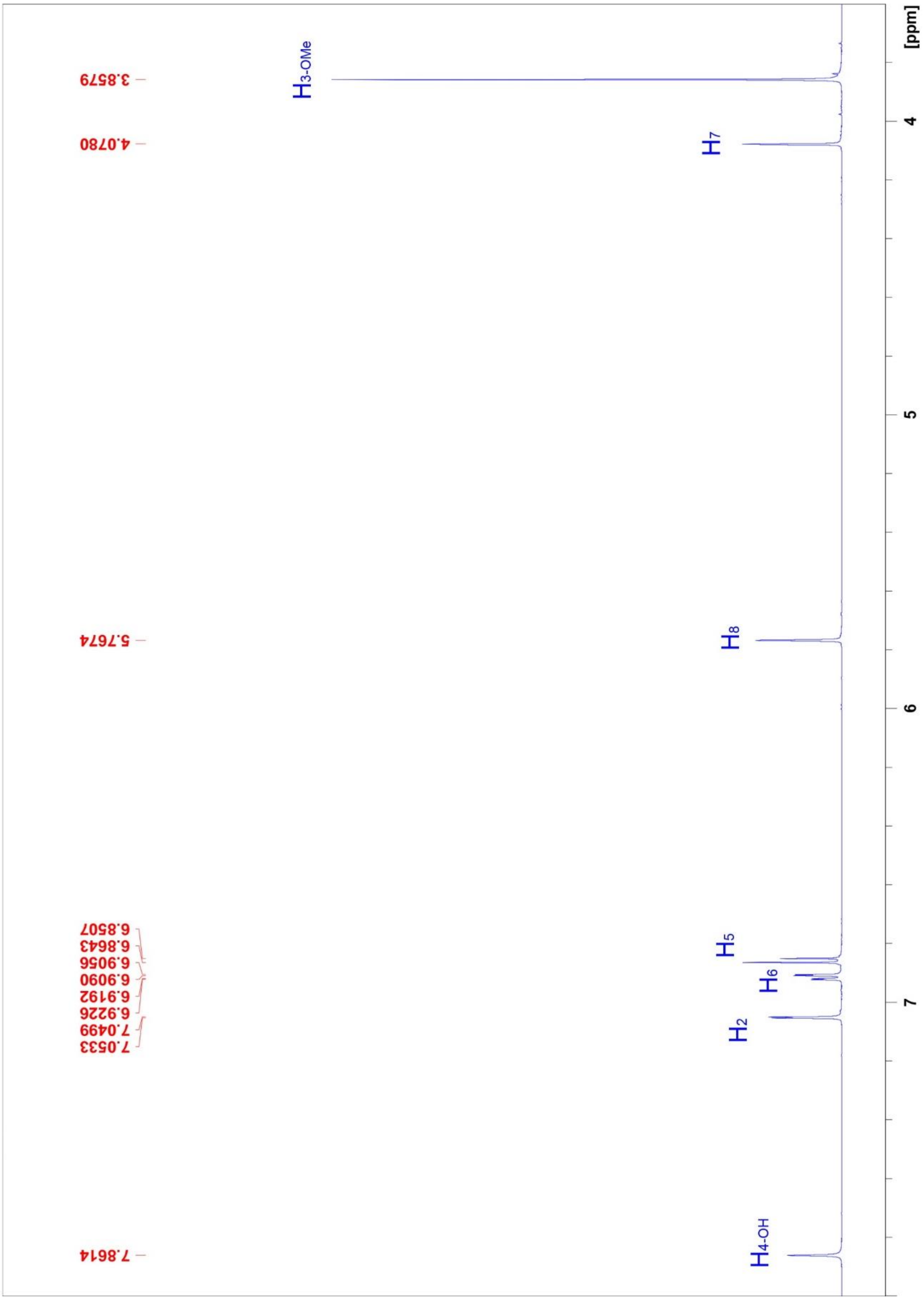

Figure S8. ^{1}H NMR spectrum of compound **1** in acetone.

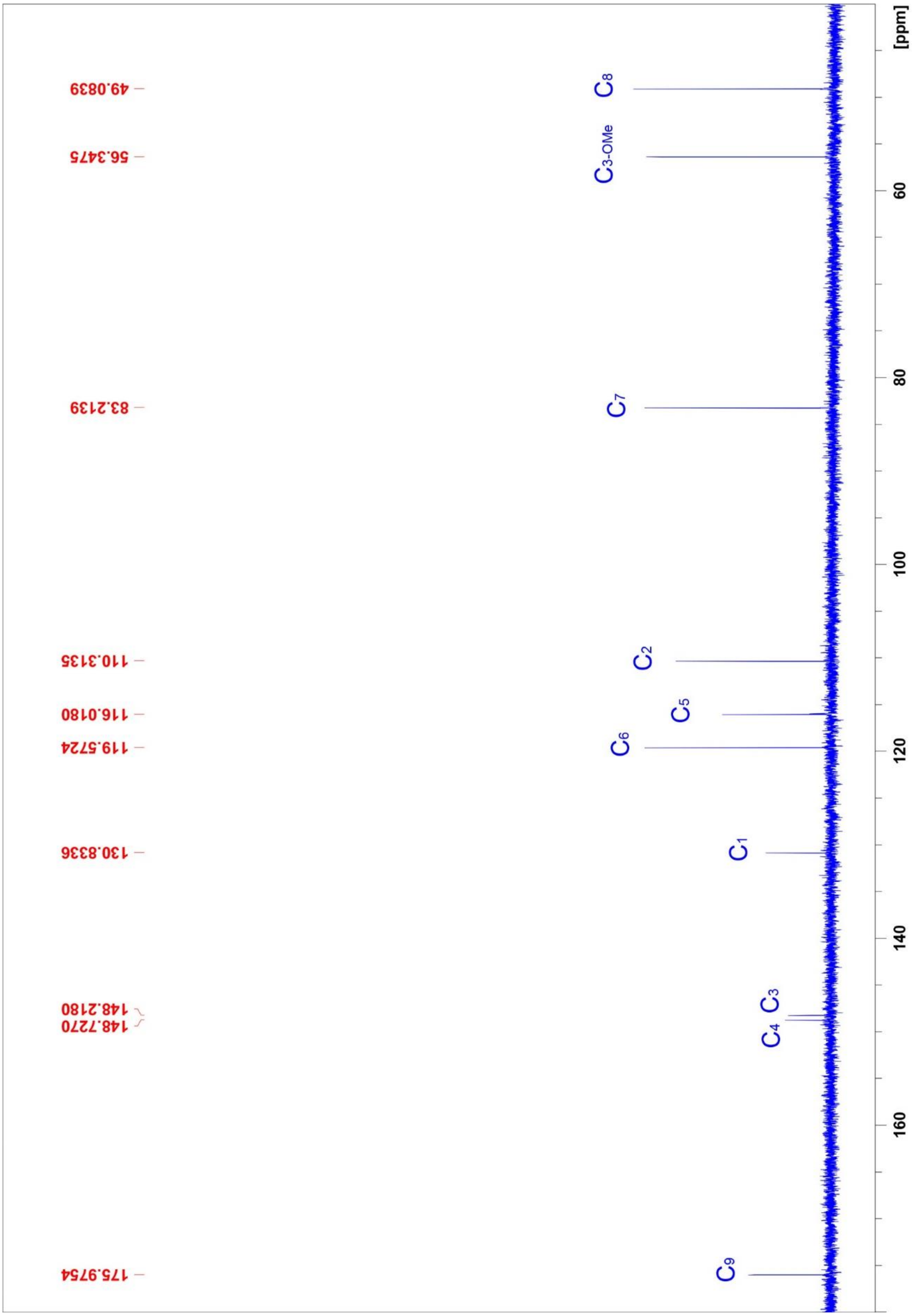

Figure S9. ^{13}C NMR spectrum of compound **1** in acetone.

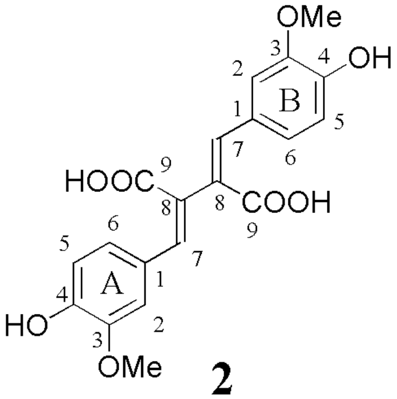

2

Table 3. ^{1}H and ^{13}C NMR data of compound **2** (Acetone d_6, 600 MHz for ^{1}H NMR and ^{13}C NMR, chemical shift δ in ppm, coupling constant *J* in Hz).

Position	δ_H (ppm)	δ_C (ppm)
3-OMe	3.74 s	56.00
1		127.89
2	7.31 (d, *J* = 2.0 Hz)	113.46
3		148.16
4		149.27
5	6.78 (d, *J* = 8.2 Hz)	115.94
6	7.12 (dd, *J* = 8.2, 2.0 Hz)	125.59
7	7.84 s	142.44
8		125.91
9		168.30

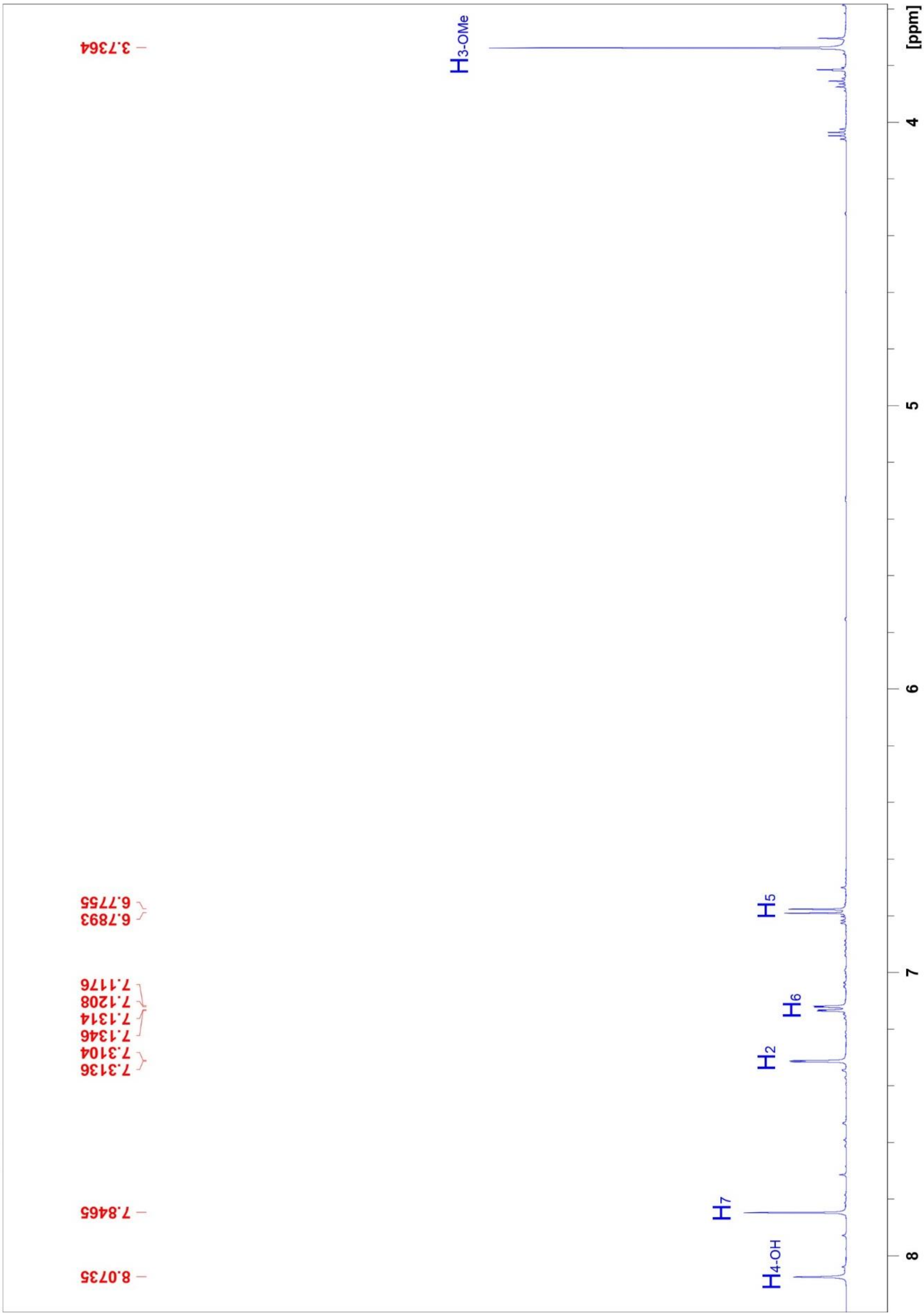

Figure S10. ^{1}H NMR spectrum of compound **2** in acetone.

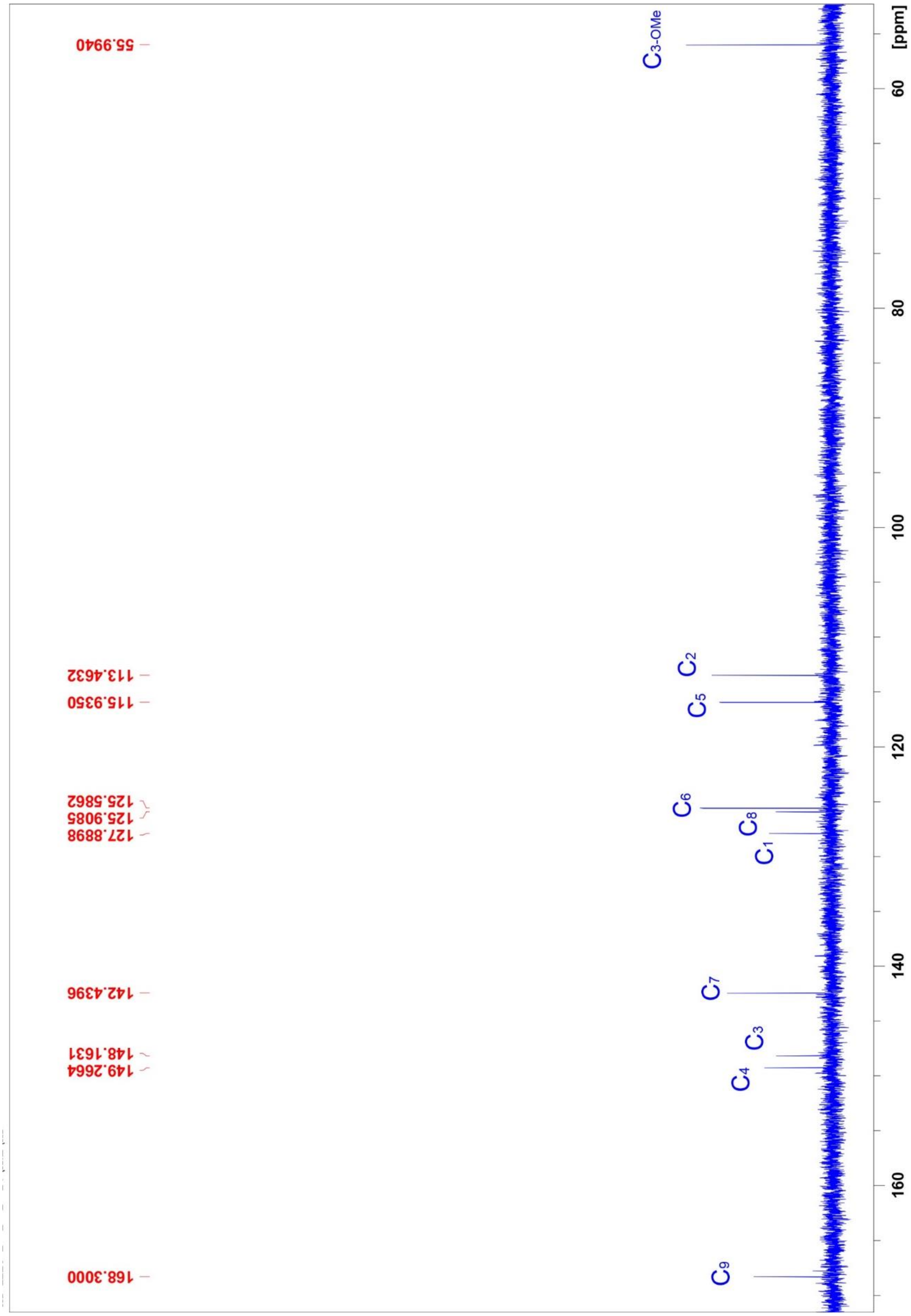

Figure S11. ^{13}C NMR spectrum of compound **2** in acetone.

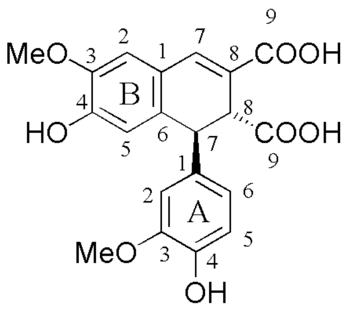

3a

Table 4. ^{1}H and ^{13}C NMR data of compound **3a** (Acetone d_6, 600 MHz for ^{1}H NMR and ^{13}C NMR, chemical shift δ in ppm, coupling constant *J* in Hz).

**Position**	**δ_H (ppm)**	**δ_C (ppm)**
A3-OMe	3.74 s	56.10
A1		135.74
A2	6.80 (d, *J* = 2.0 Hz)	111.82
A3		148.07
A4		146.07
A5	6.64 (d, *J* = 8.2 Hz)	115.46
A6	6.42 (dd, *J* = 8.2, 2.0 Hz)	120.60
A7	4.61 (br d, *J* = 1.8 Hz)	46.09
A8	3.96 (d, *J* = 1.9 Hz)	47.65
A9		173.70
B3-OMe	3.87 s	56.30
B1		132.20
B2	7.10 s	113.04
B3		147.49
B4		149.42
B5	6.73 s	116.88
B6		124.55
B7	7.70 s	137.35
B8		123.51
B9		168.67

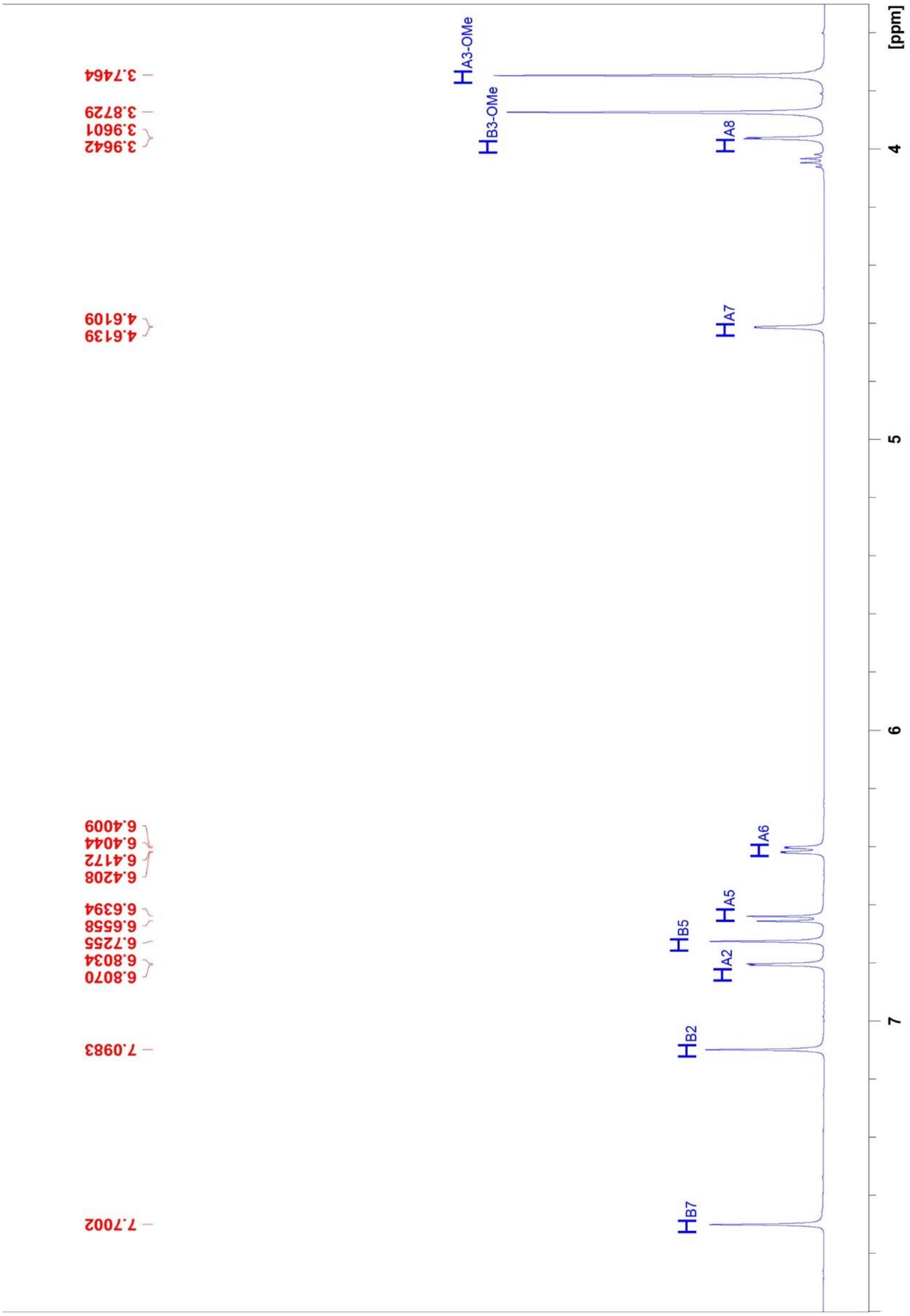

Figure S12. ^{1}H NMR spectrum of compound **3a** in acetone.

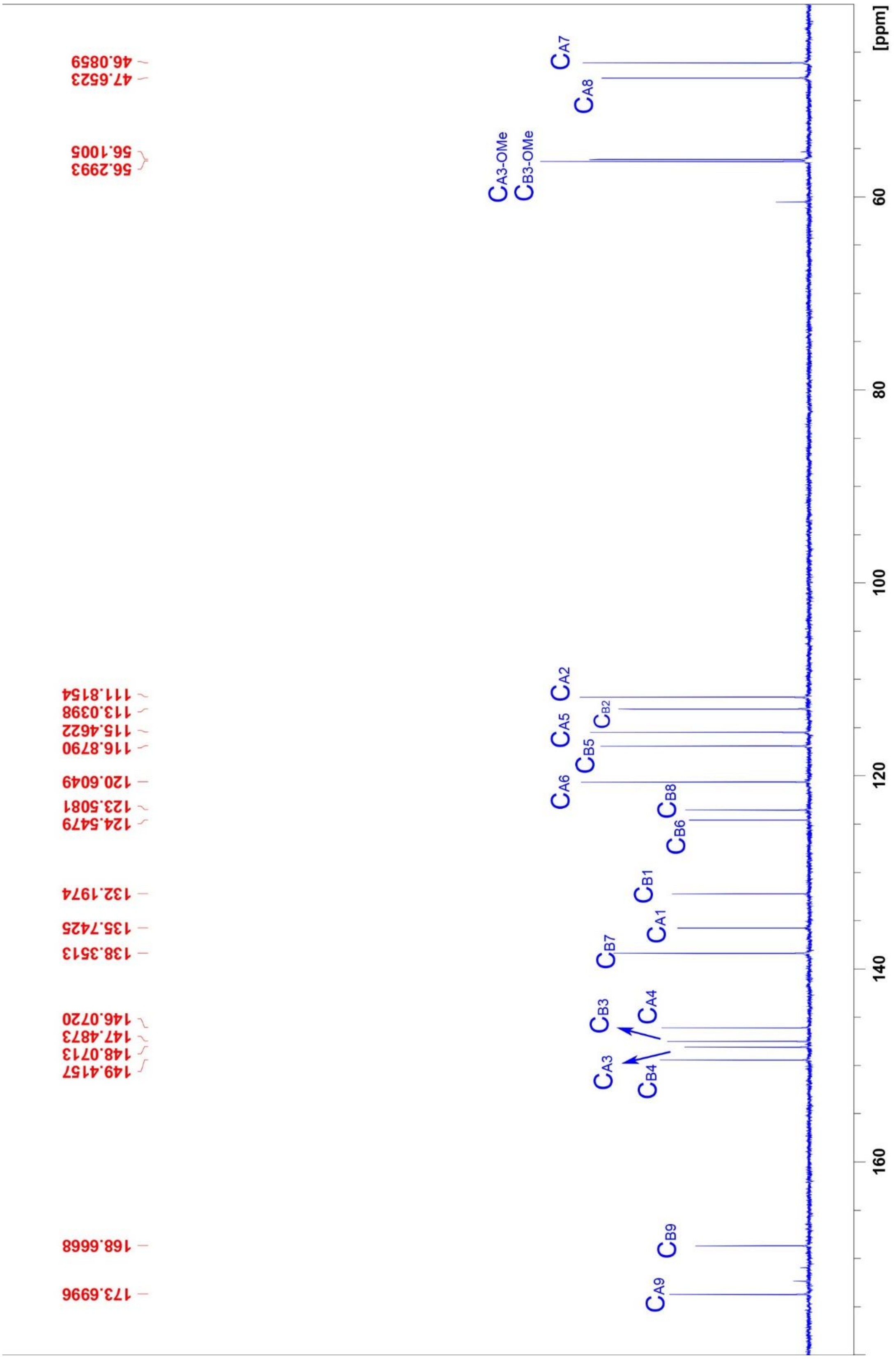

Figure S13. ^{13}C NMR spectrum of compound **3a** in acetone.

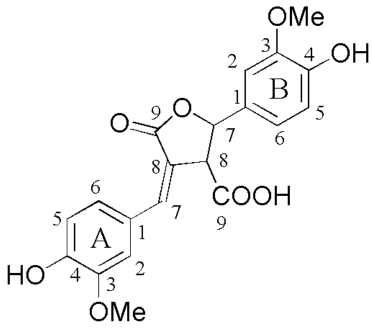

4

Table 5. ^{1}H and ^{13}C NMR data of compound **4** (Acetone d$_6$, 600 MHz for ^{1}H NMR and ^{13}C NMR, chemical shift δ in ppm, coupling constant *J* in Hz).

**Position**	**δH (ppm)**	**δC (ppm)**
A3-OMe	3.86 s	56.20
A1		126.52
A2	7.39 (d, *J* = 2.0 Hz)	113.84
A3		148.45
A4		150.07
A5	6.89 (d, *J* = 8.2 Hz)	116.12
A6	7.21 (dd, *J* = 8.2, 2.0 Hz)	126.57
A7	7.60 (br d, *J* = 2.0 Hz)	140.37
A8		120.49
A9		171.70
B3-OMe	3.81 s	56.19
B1		132.36
B2	6.90 s	110.07
B3		148.53
B4		147.84
B5	6.88 s	115.96
B6	6.82 (d, *J* = 2.0 Hz)	119.10
B7	5.75 (d, *J* = 2.8 Hz)	81.25
B8	4.30 (t, *J* = 2.5 Hz)	54.08
B9		172.32

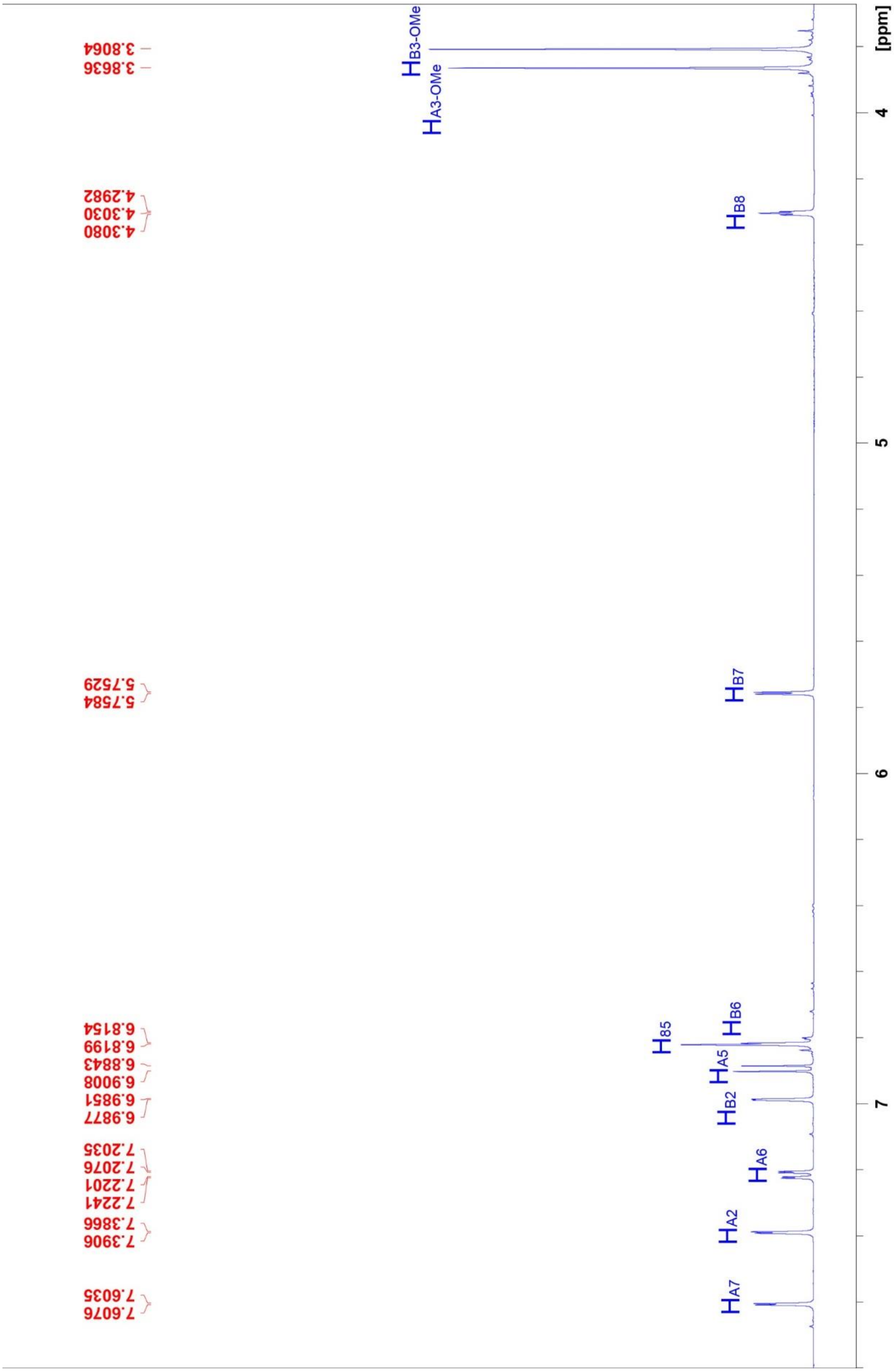

Figure S14. ^{1}H NMR spectrum of compound **4** in acetone.

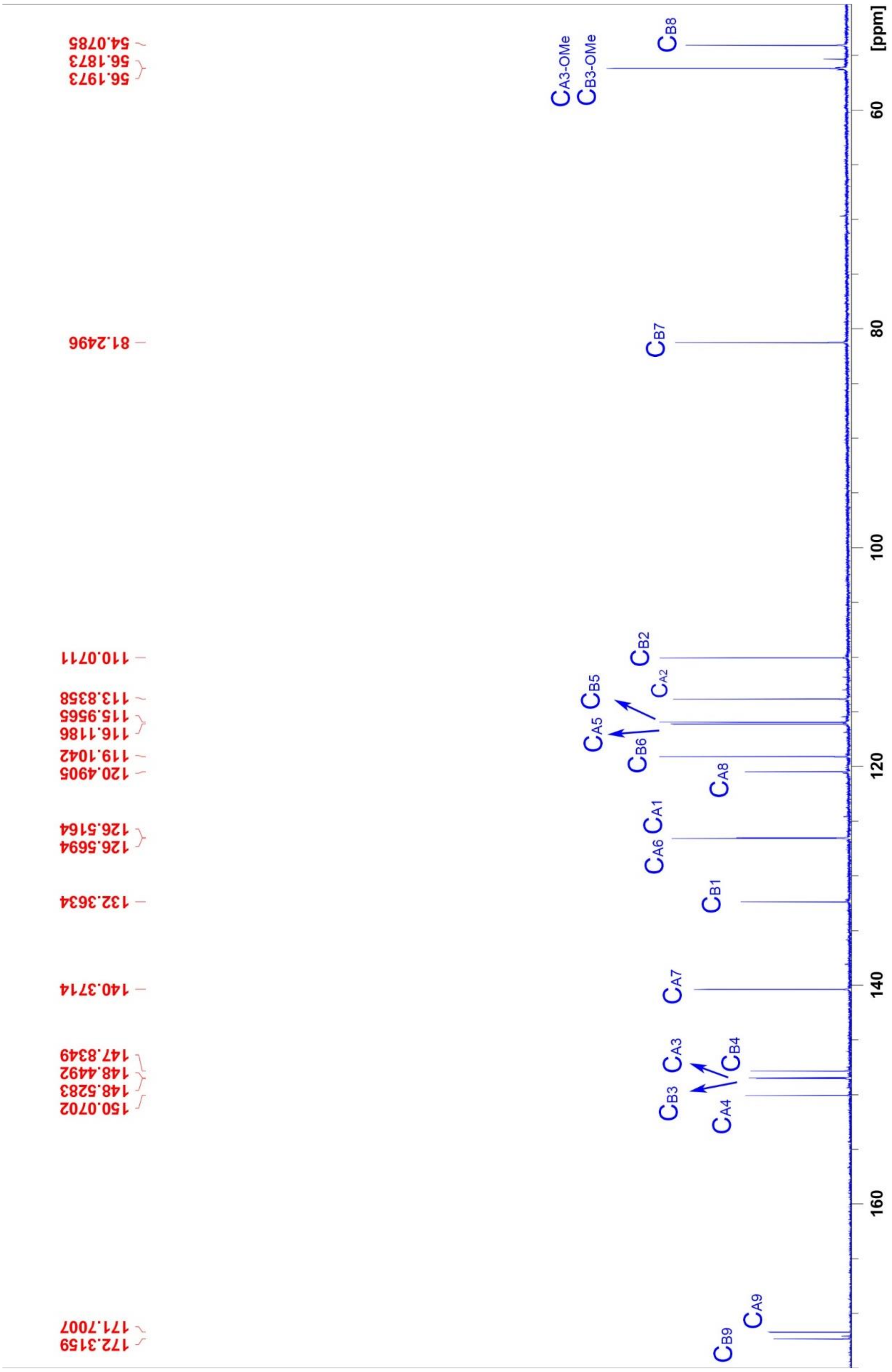

Figure S15. ^{13}C NMR spectrum of compound **4** in acetone.

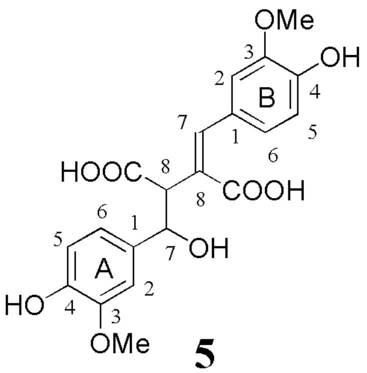

5

Table 6. ^{1}H and ^{13}C NMR data of compound **5** (Acetone d_6, 600 MHz for ^{1}H NMR and ^{13}C NMR, chemical shift δ in ppm, coupling constant *J* in Hz).

Position	δ_H (ppm)	δ_C (ppm)
A3-OMe	3.67 s	55.90
A1		135.82
A2	6.79 (d, *J* = 1.9 Hz)	110.81
A3		147.75
A4		146.39
A5	6.67 (d, *J* = 8.1 Hz)	114.99
A6	6.76 (dd, *J* = 8.0, 1.8 Hz)	119.92
A7	5.38 (d, *J* = 6.5 Hz)	73.15
A8	4.40 (d, *J* = 6.4 Hz)	52.70
A9		172.69
B3-OMe	3.80 s	56.20
B1		148.15
B2	6.87 (d, 6.9)	113.24
B3		148.19
B4		144.58
B5	6.81 (d, *J* = 8.1 Hz)	115.68
B6	6.83 (dd, *J* = 6.8, 2.1 Hz)	123.15
B7	7.78 s	127.72
B8		127.91
B9		171.84

Compound **5**, high resolution mass spectrum (HRMS): exact mass calculated for $[M + Na]^+$ ($C_{20}H_{20}O_9$) requires m/z 427.1005 and was found as m/z 427.1000.

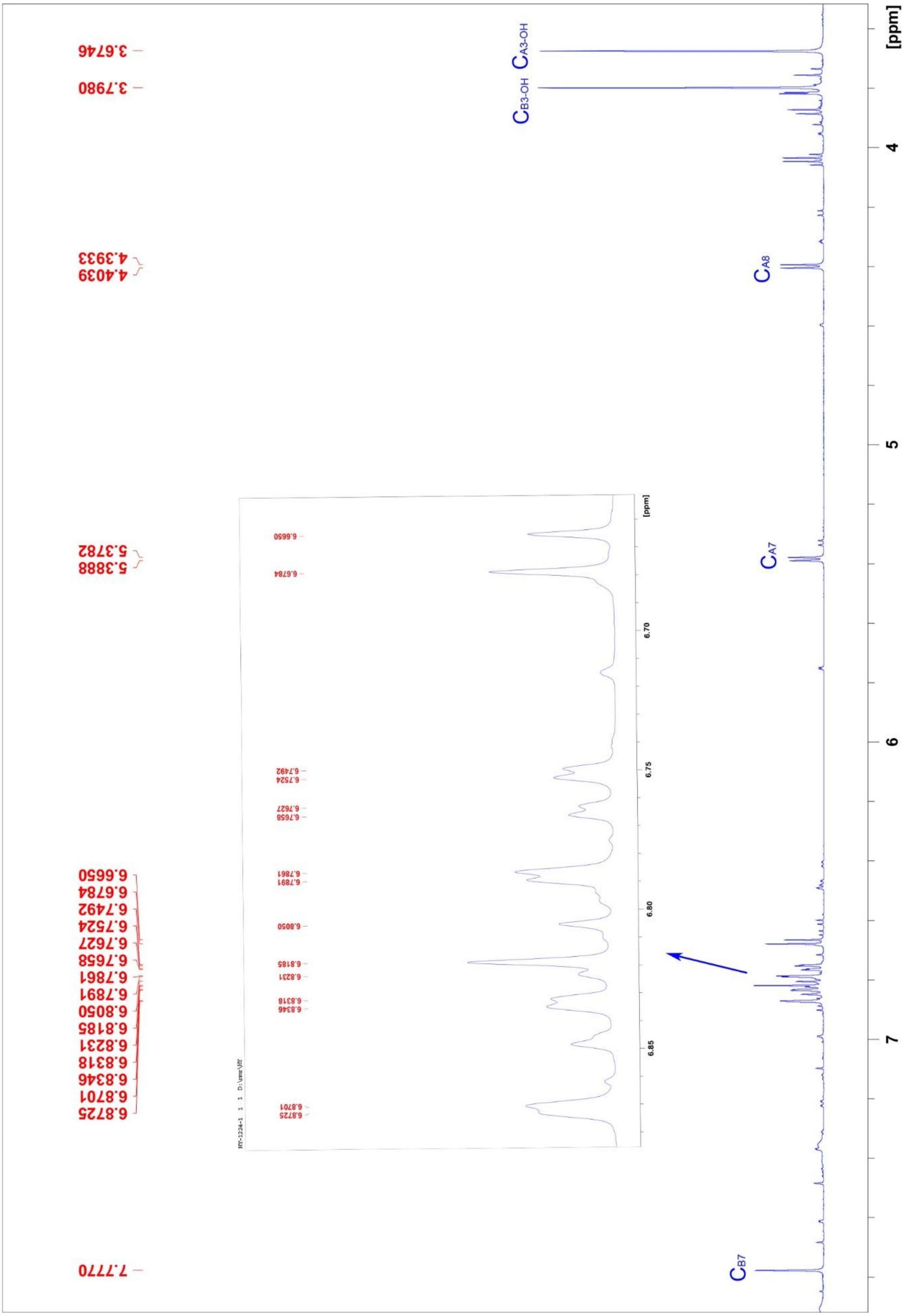

Figure S16. ^{1}H NMR spectrum of compound **5** in acetone.

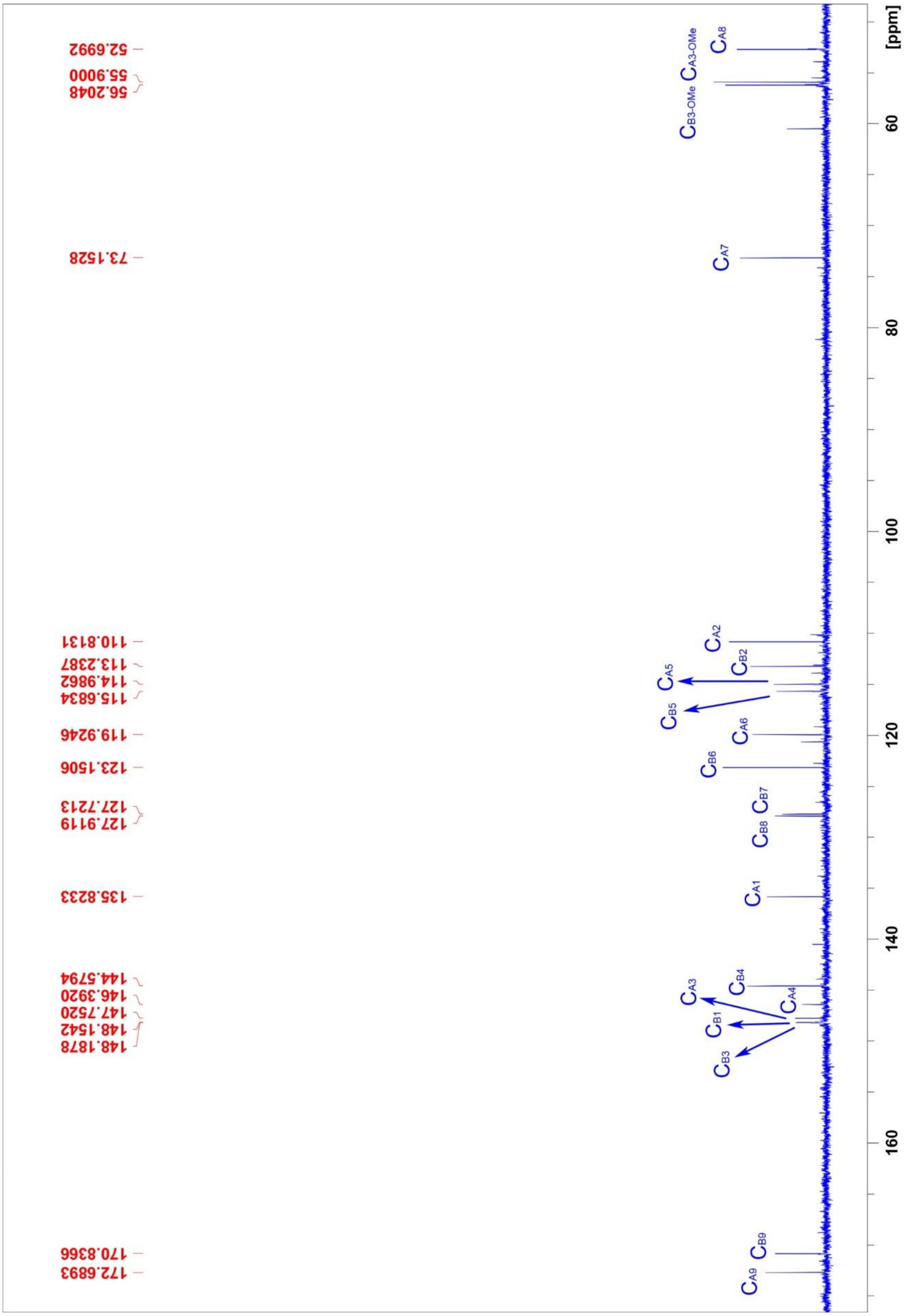

Figure S17. ^{13}C NMR spectrum of compound **5** in acetone.

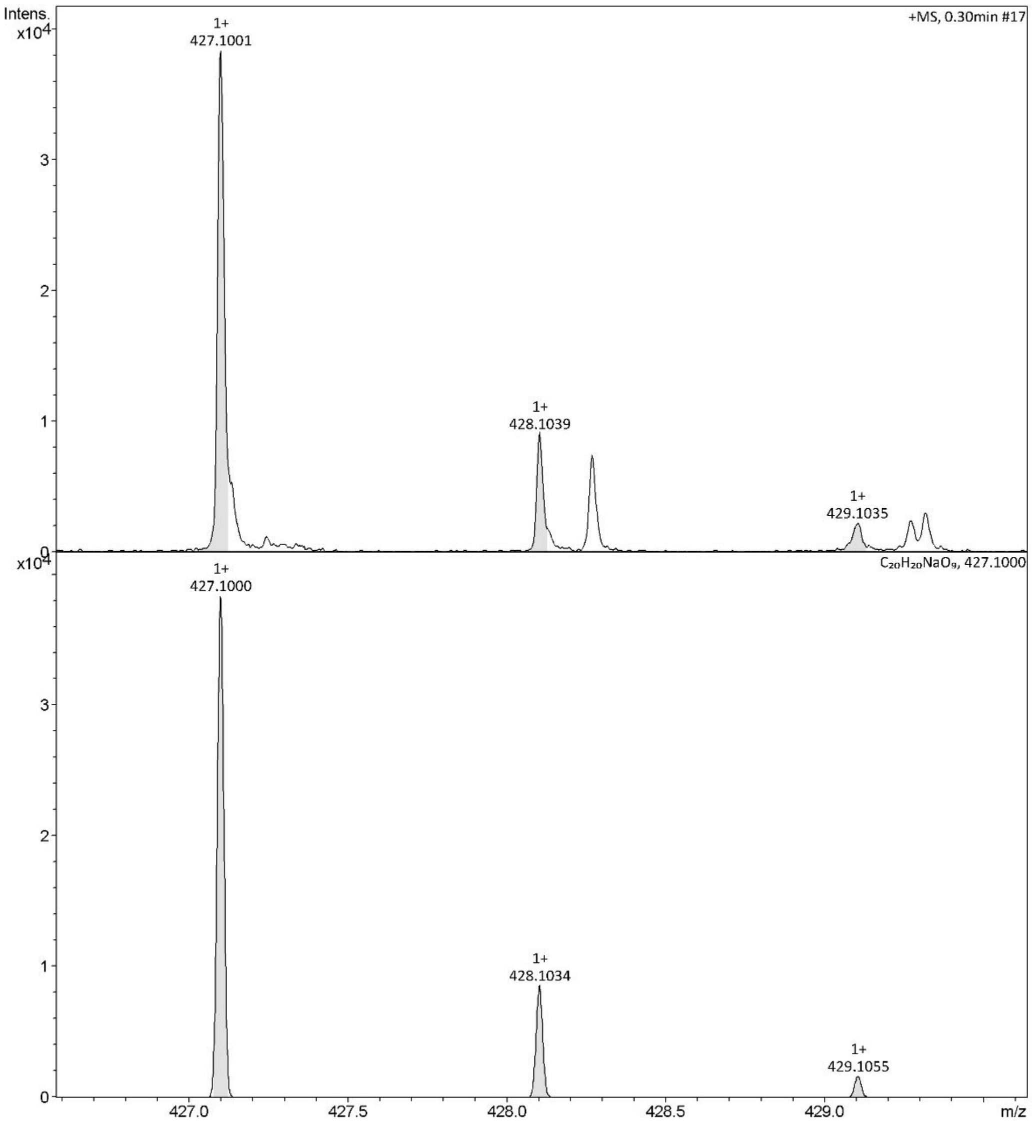

Figure S18. HRMS of compound **5**.

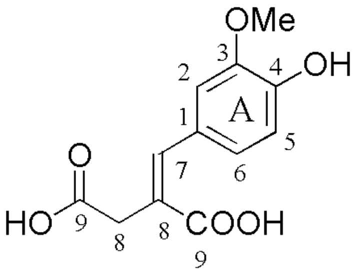

6

Table 7. ^1H and ^{13}C NMR data of compound **6** (Acetone d_6, 600 MHz for ^1H NMR and ^{13}C NMR, chemical shift δ in ppm, coupling constant *J* in Hz).

Position	δ_H (ppm)	δ_C (ppm)
A3-OMe	3.88 s	56.23
A1		142.36
A2	7.15 (d, *J* = 1.9 Hz)	124.84
A3		148.70
A4		148.37
A5	6.92 (d, *J* = 8.1 Hz)	116.07
A6	7.02 (dd, *J* = 8.2, 1.8 Hz)	127.77
A7	7.84 s	124.16
A8		34.10
A9		172.70
B8	3.61 s	113.74
B9		169.41

Compound **6,** HRMS: exact mass calculated for $[M + Na]^+$ ($C_{12}H_{12}O_6$) requires m/z 275.0532 and was found as m/z 275.0526.

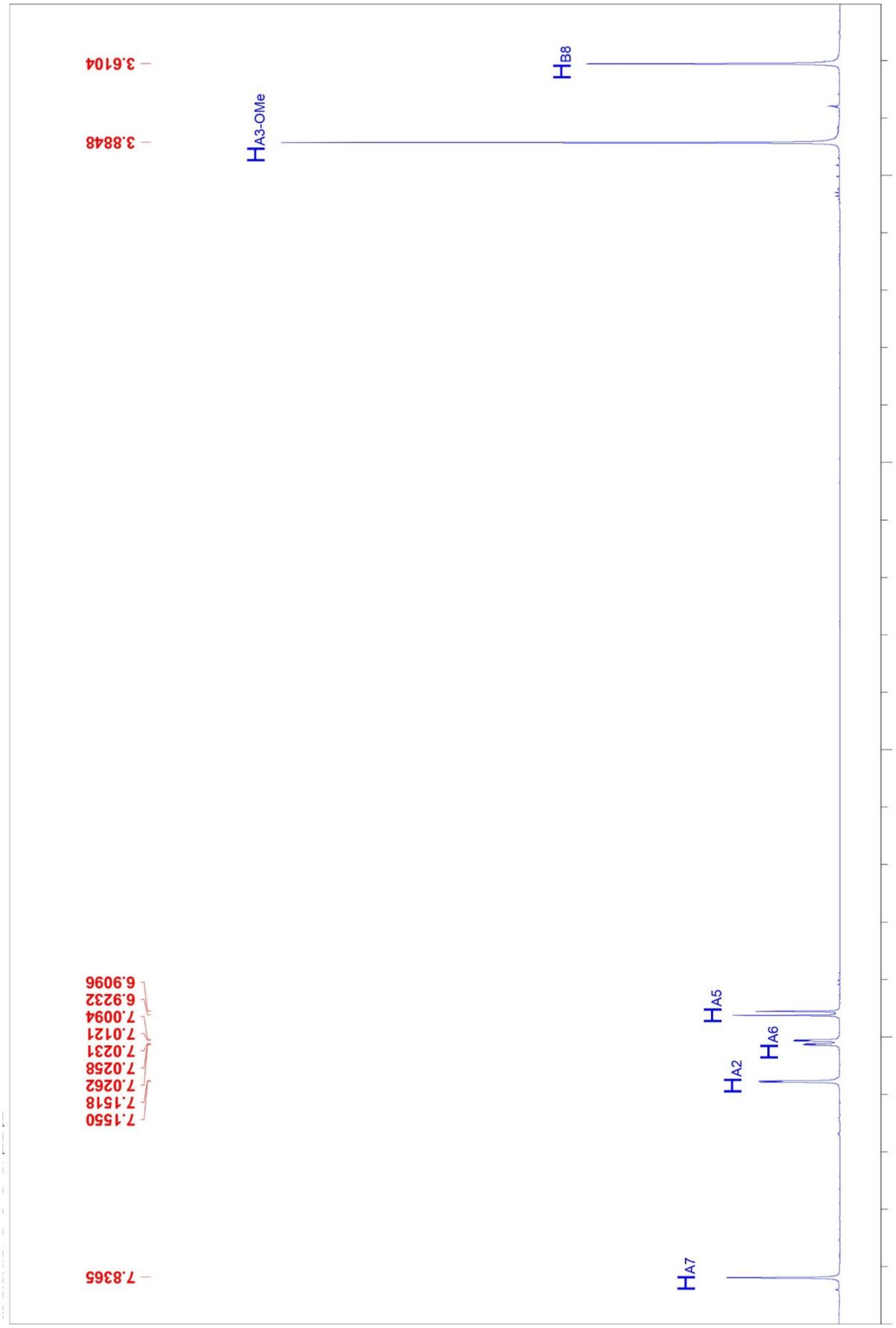

Figure S19. ^{1}H NMR spectrum of compound **6** in acetone.

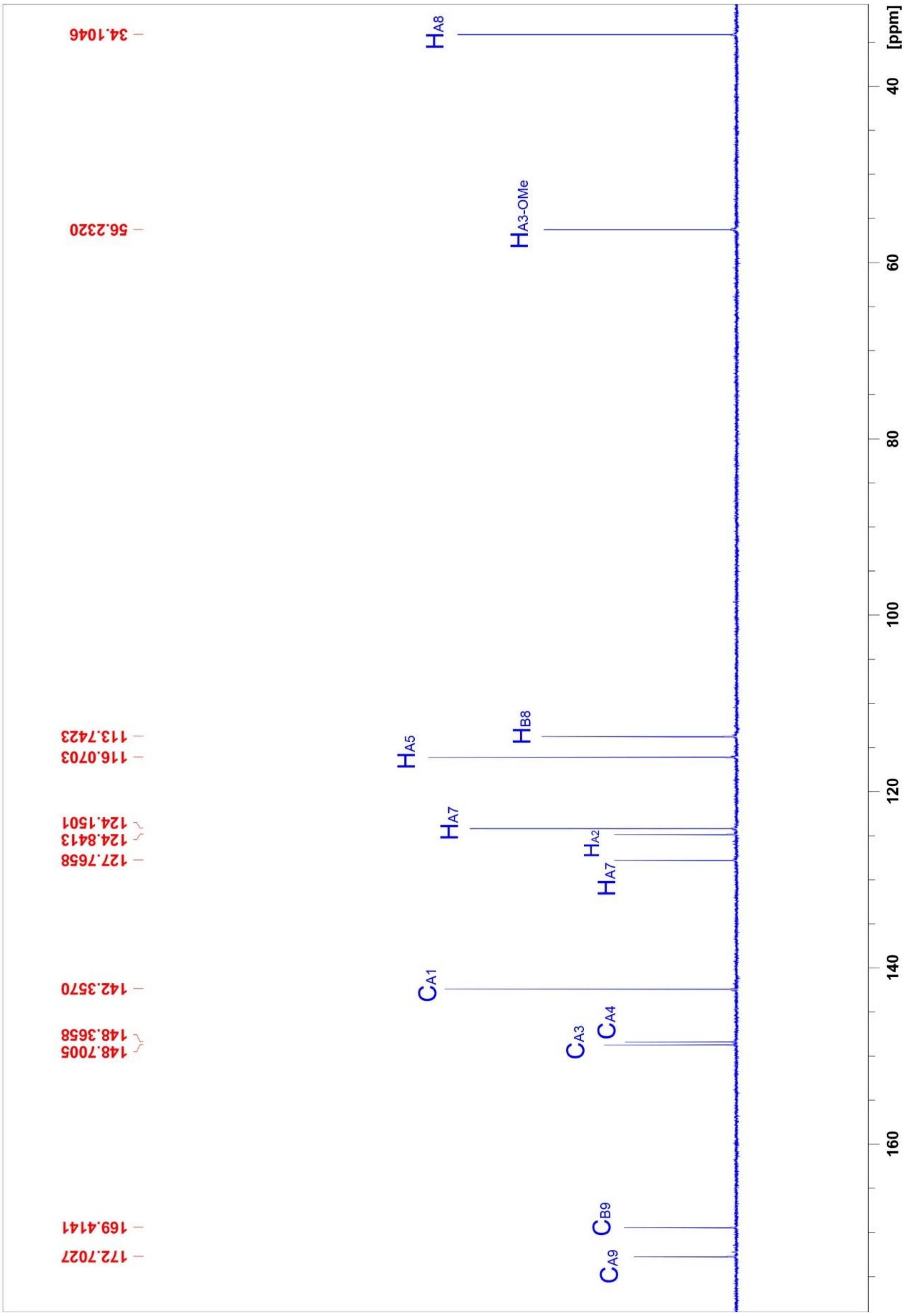

Figure S20. ^{13}C NMR spectrum of compound **6** in acetone.

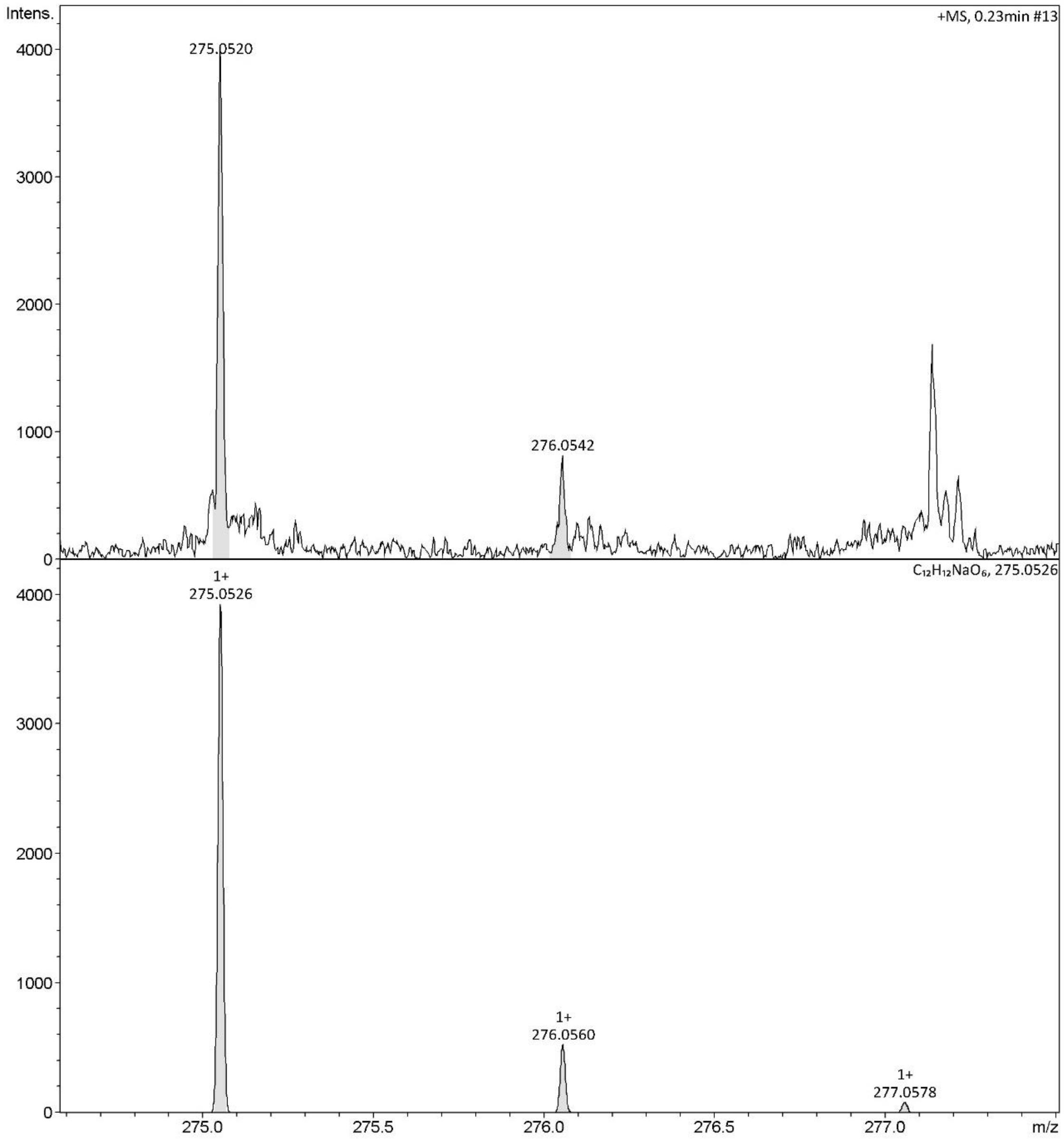

Figure S21. HRMS of compound **6**.

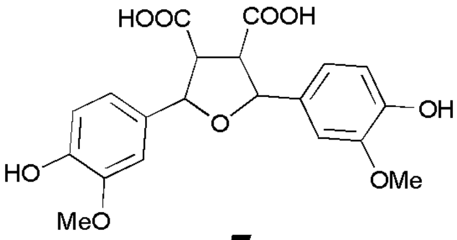

7

Table 8. ^{1}H and ^{13}C NMR data of compound **7** (Acetone d_6, 600 MHz for ^{1}H NMR and ^{13}C NMR, chemical shift δ in ppm, coupling constant *J* in Hz).

Position	δ_H (ppm)	δ_C (ppm)
3-OMe	3.84 s	56.22
1		133.34
2	7.12 (d, *J* = 1.8 Hz)	110.57
3		148.36
4		147.28
5	6.83 (d, *J* = 8.2 Hz)	115.59
6	6.94 (dd, *J* = 8.0, 1.7 Hz)	119.91
7	5.33 (dd, *J* = 5.7, 2.5 Hz)	84.13
8	3.55 (dd, *J* = 5.7, 2.5 Hz)	57.73
9		172.82

Compound **7,** HRMS: exact mass calculated for $[M + Na]^+$ ($C_{20}H_{20}O_9$) requires m/z 427.1005 and was found as m/z 427.1000.

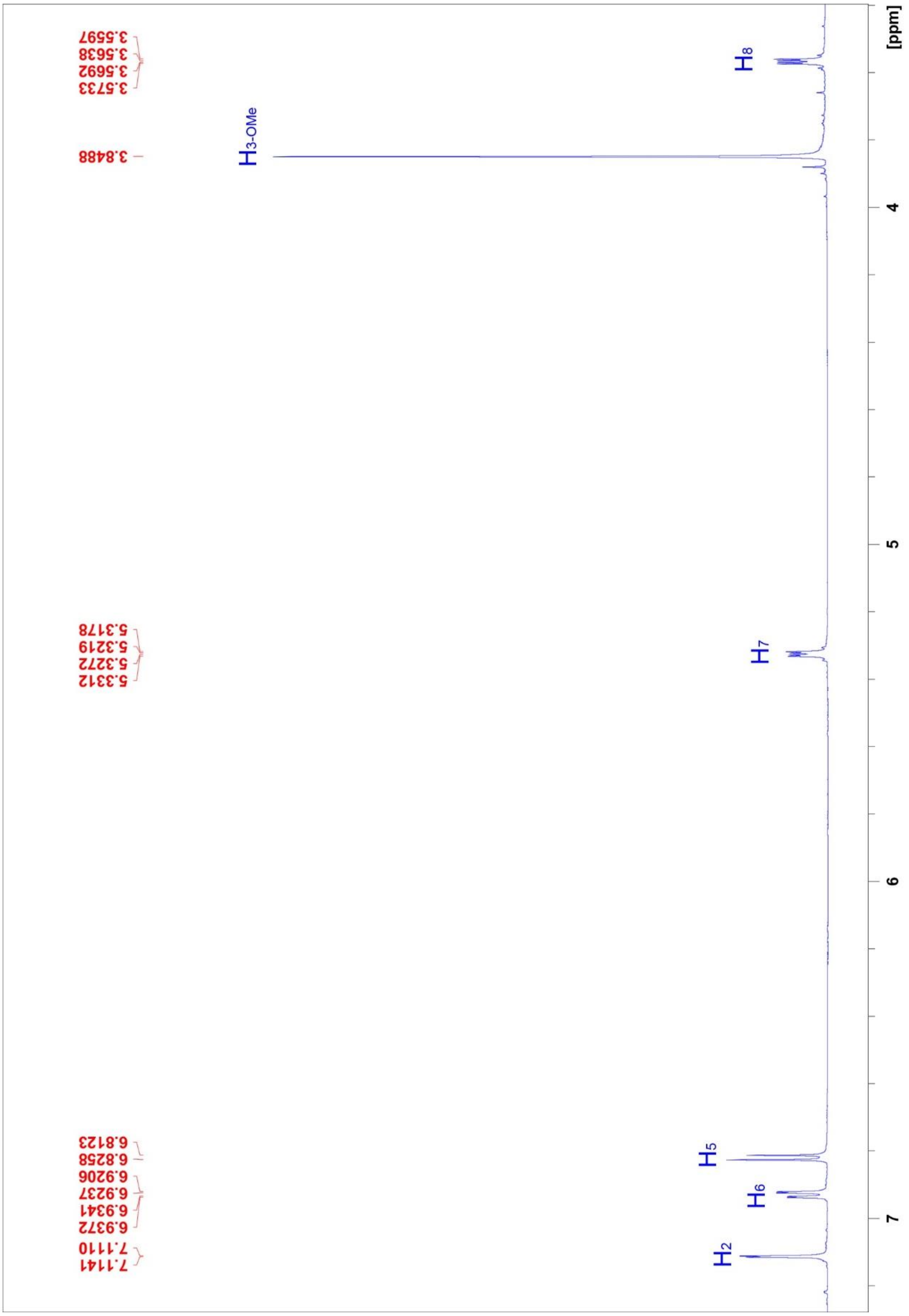

Figure S22. ^{1}H NMR spectrum of compound **7** in acetone.

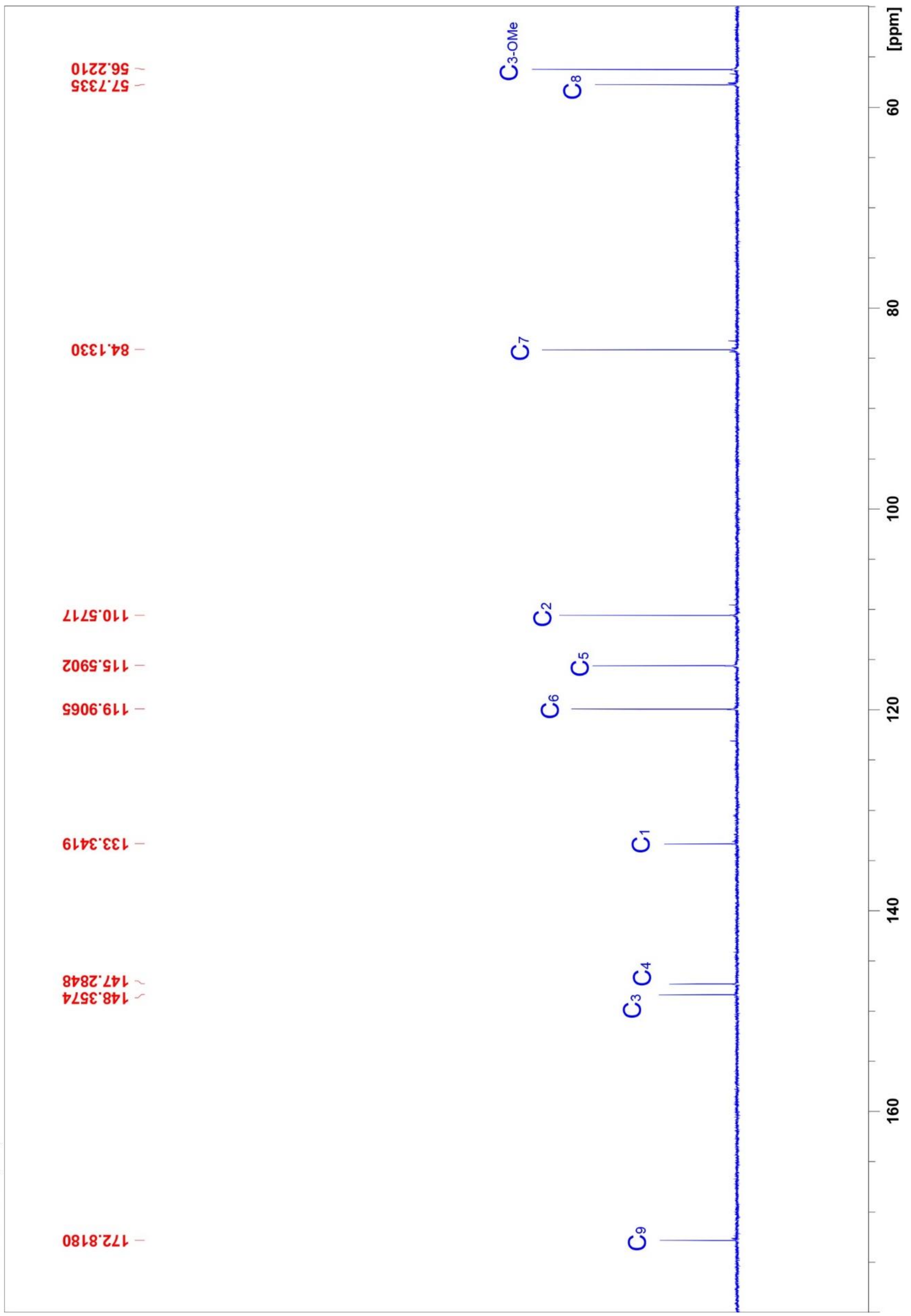

Figure S23. ^{13}C NMR spectrum of compound **7** in acetone.

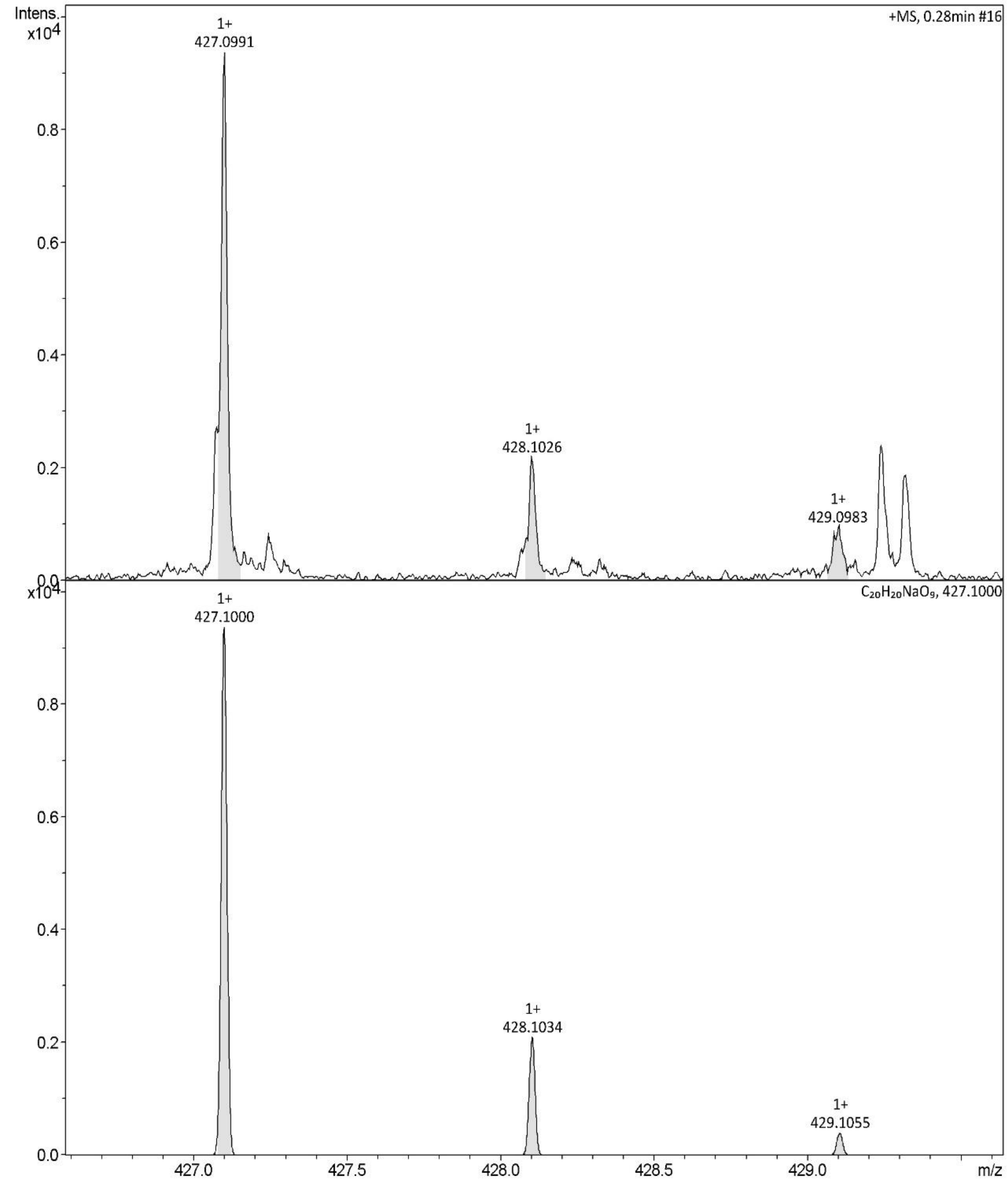

Figure S24. HRMS of compound **7**.

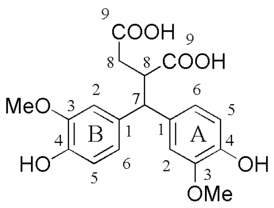

8

Table 9. ^{1}H and ^{13}C NMR data of compound **8** (Acetone d_6, 600 MHz for ^{1}H NMR and ^{13}C NMR, chemical shift δ in ppm, coupling constant *J* in Hz).

**Position**	**δ_H (ppm)**	**δ_C (ppm)**
A3-OMe	3.68 s	56.26
A1		134.97
A2	6.92 (d, *J* = 2.0 Hz)	112.40
A3		148.38
A4		146.11
A5	6.56 (d, *J* = 8.1 Hz)	115.78
A6	6.71 (dd, *J* = 8.0, 2.0 Hz)	121.43
A8	2.26 (dd, *J* = 16.5, 3.30 Hz); 2.47 (dd, *J* = 16.7, 10.8 Hz)	47.07
A9		175.44
B3-OMe	3.72 s	56.22
B1		135.34
B2	6.93 (d, *J* = 2.0 Hz)	112.57
B3		147.89
B4		145.94
B5	6.63 (d, *J* = 8.1 Hz)	115.35
B6	6.73 (dd, *J* = 8.1, 2.0 Hz)	121.43
B7	3.84 (d, *J* = 11.4 Hz)	54.44
B8	3.65 (td, *J* = 11.4, 3.4 Hz)	36.78
B9		173.11

Compound **8**, HRMS: exact mass calculated for $[M + Na]^+$ ($C_{19}H_{20}O_8$) requires m/z 399.1056 and was found as m/z 399.1050.

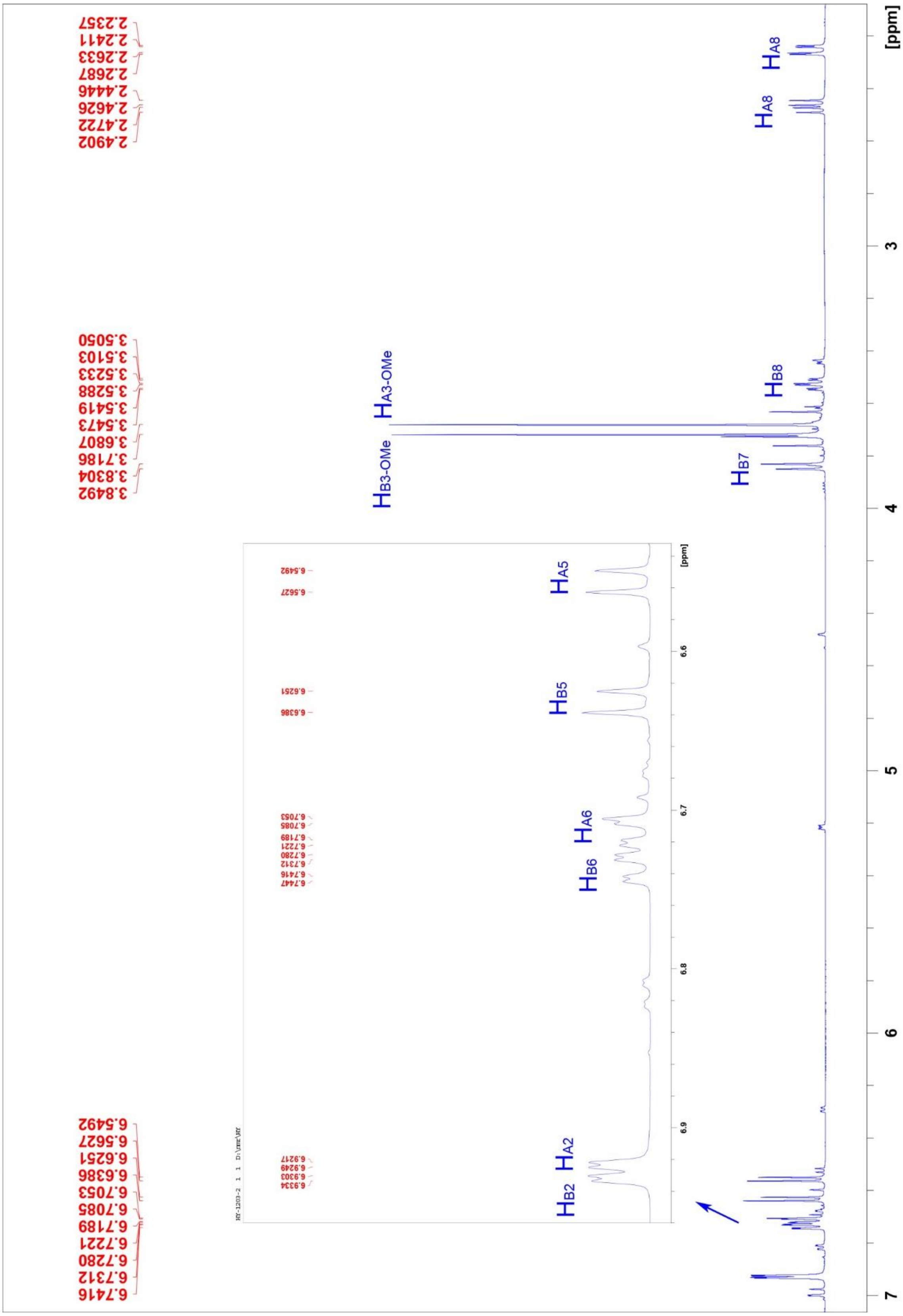

Figure S25. ^{1}H NMR spectrum of compound **8** in acetone.

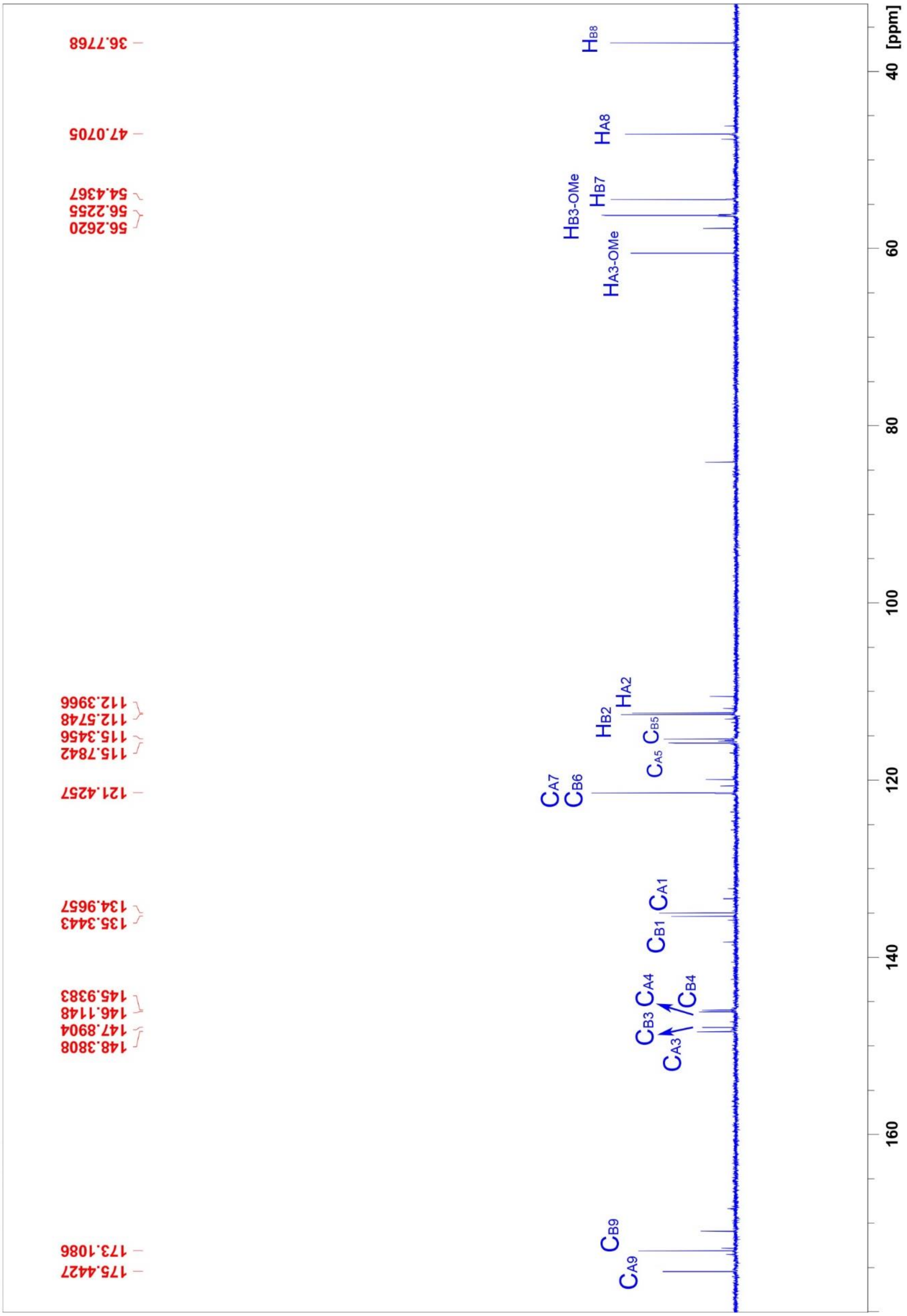

Figure S26. ^{13}C NMR spectrum of compound **8** in acetone.

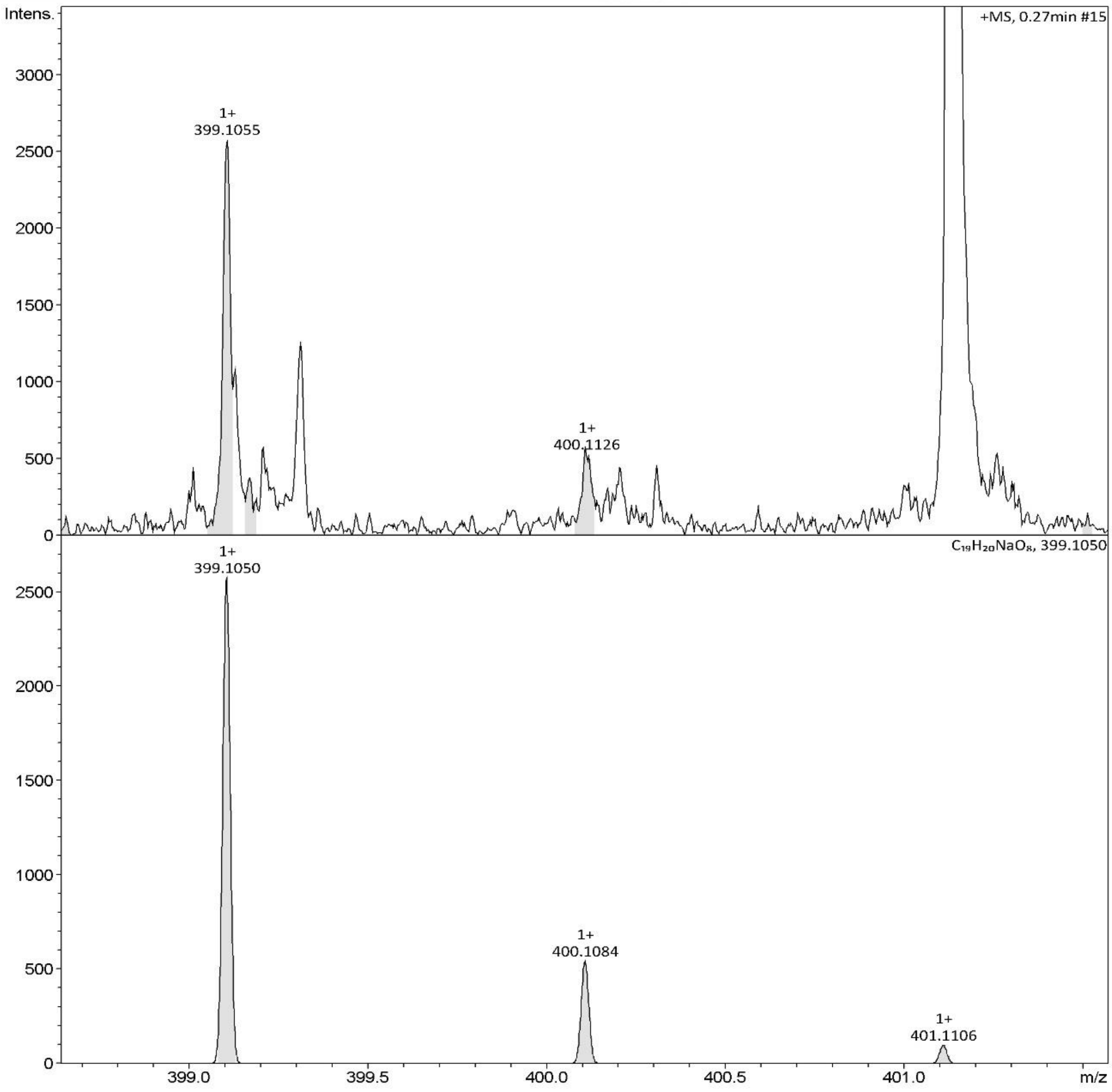

Figure S27. HRMS of compound **8**.

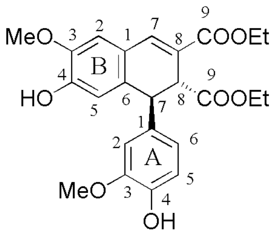

9a

Table 10. ^{1}H and ^{13}C NMR data of compound **9a** (Acetone d_6, 600 MHz for ^{1}H NMR and ^{13}C NMR, chemical shift δ in ppm, coupling constant *J* in Hz).

**Position**	**δ_H (ppm)**	**δ_C (ppm)**
A3-OMe	3.74 s	56.14
A1		135.29
A2	6.78 (d, *J* = 2.0 Hz)	111.98
A3		148.12
A4		146.22
A5	6.64 (d, *J* = 8.2 Hz)	115.52
A6	6.42 (dd, *J* = 8.2, 2.0 Hz)	120.91
A7	4.51 (d, *J* = 2.0 Hz)	46.42
A8	3.99 (d, *J* = 1.9 Hz)	48.24
A9		172.71
B3-OMe	3.98 s	56.31
B1		132.11
B2	7.09 s	113.08
B3		147.49
B4		149.43
B5	6.65 s	116.70
B6	6.86 (dd, *J* = 8.1, 2.0 Hz)	123.59
B7	7.64 s	137.97
B8		124.39
B9		167.03

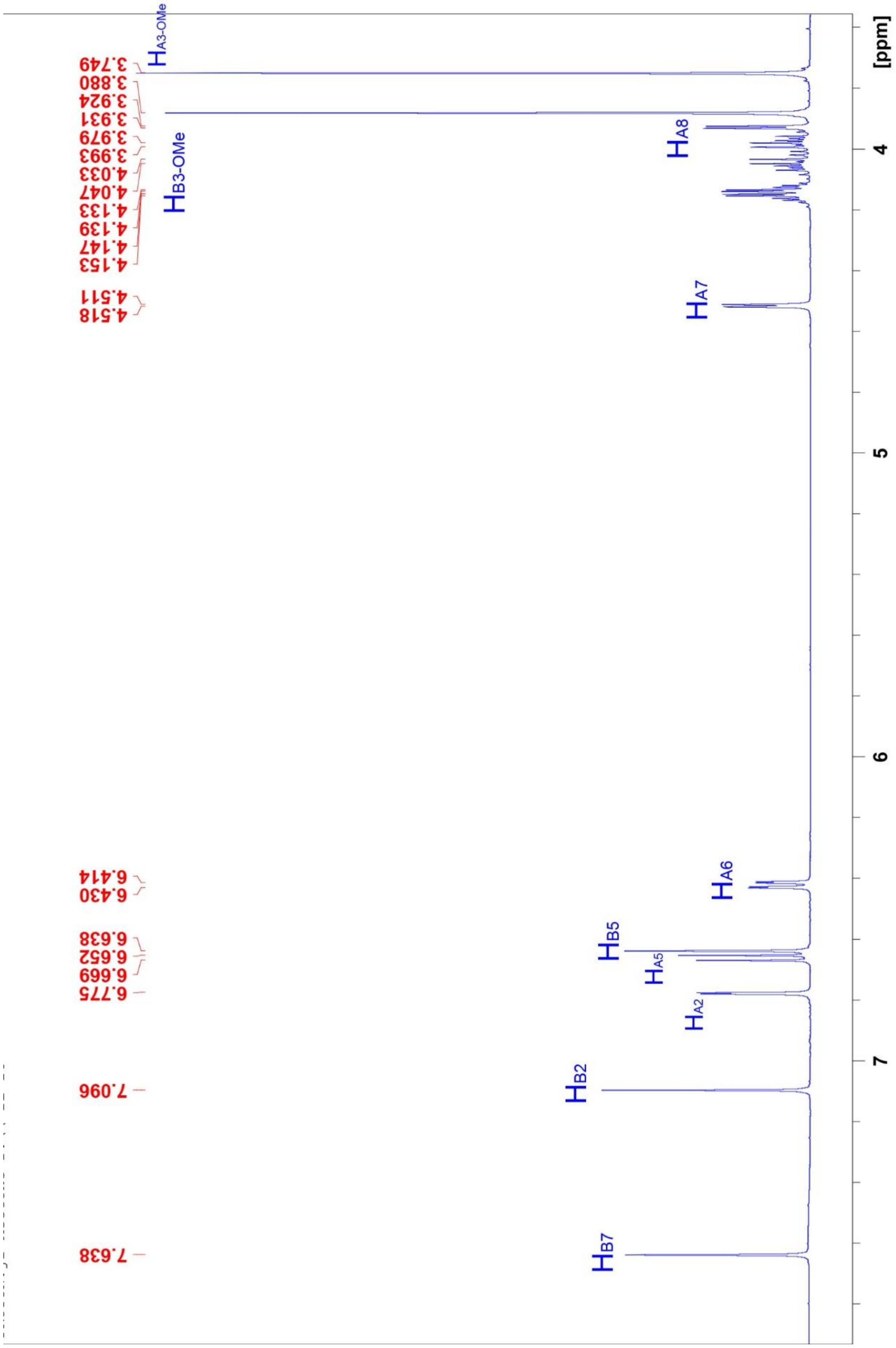

Figure S28. ^{1}H NMR spectrum of compound **9a** in acetone.

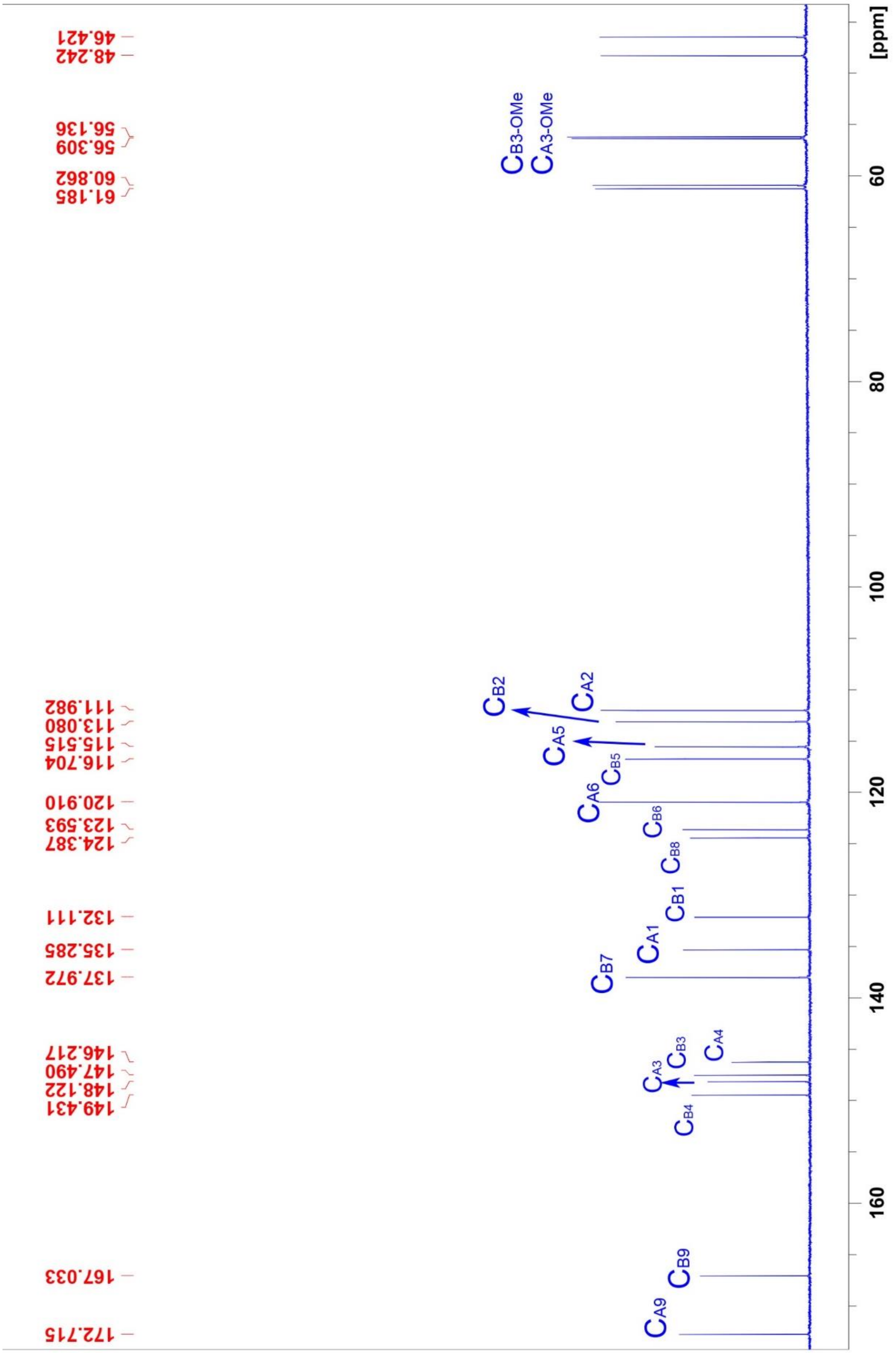

Figure S29. ^{13}C NMR spectrum of compound **9a** in acetone.

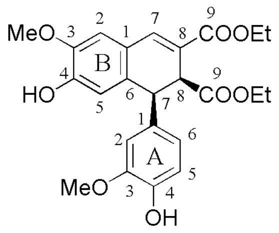

9b

Table 11. ^{1}H and ^{13}C NMR data of compound **9b** (Acetone d$_6$, 600 MHz for ^{1}H NMR and ^{13}C NMR, chemical shift δ in ppm, coupling constant *J* in Hz).

Position	δH (ppm)	δC (ppm)
A3-OMe	3.78 s	56.32
A1		132.90
A2	6.85 (d, *J* = 2.0 Hz)	113.94
A3		148.11
A4		146.82
A5	6.80 (d, *J* = 8.1 Hz)	115.67
A6	6.72 (dd, *J* = 8.2, 2.0 Hz)	123.91
A7	4.49 (d, *J* = 8.25 Hz)	47.95
A8	3.86 (d, *J* = 8.4 Hz)	48.09
A9		171.83
B3-OMe	3.88 s	56.54
B1		132.22
B2	7.06 s	114.05
B3		147.09
B4		149.30
B5	6.52 s	116.07
B6		125.00
B7	7.59 s	138.80
B8		125.36
B9		167.15

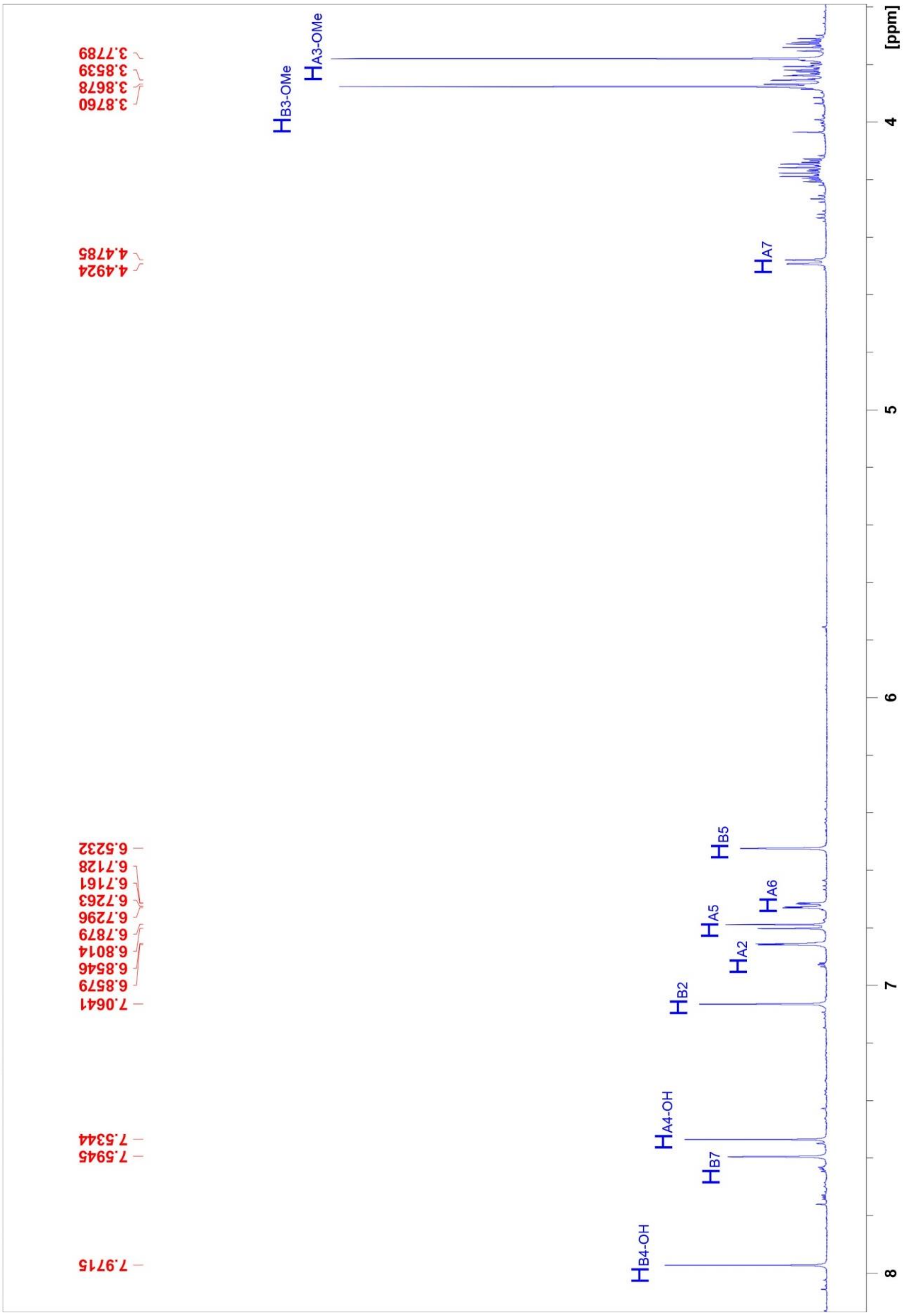

Figure S30. ^{1}H NMR spectrum of compound **9b** in acetone

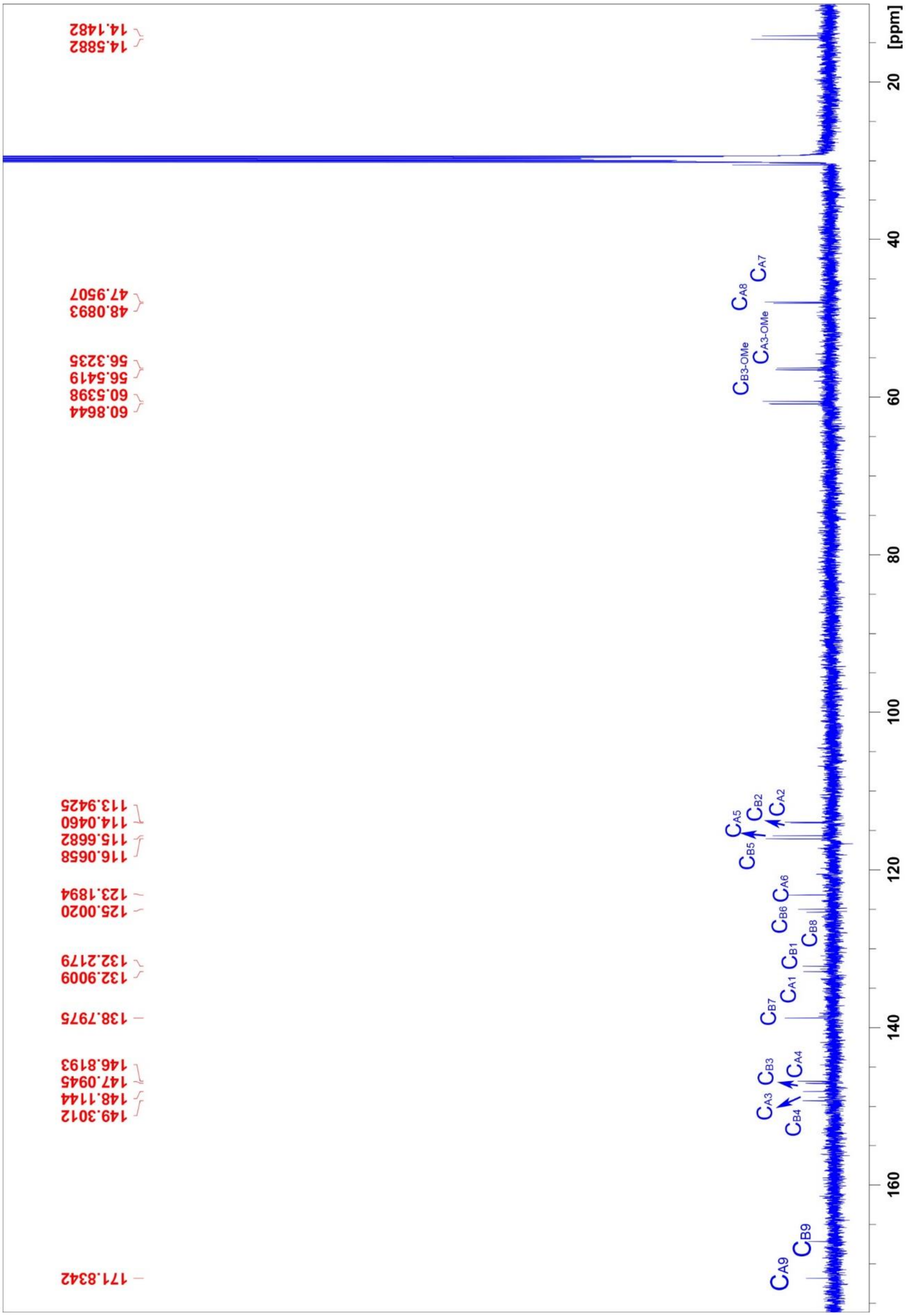

Figure S31. ^{13}C NMR spectrum of compound **9b** in acetone.

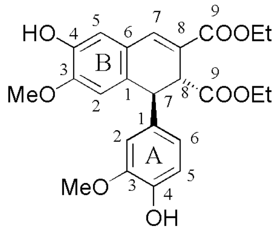

10

Table 12. ^{1}H and ^{13}C NMR data of compound **10** (Acetone d_6, 600 MHz for ^{1}H NMR and ^{13}C NMR, chemical shift δ in ppm, coupling constant *J* in Hz).

Position	δ_H (ppm)	δ_C (ppm)
A3-OMe	3.74 s	56.12
A1		135.53
A2	6.74 (d, *J* = 2.0 Hz)	111.89
A3		148.12
A4		146.55
A5	6.66 (d, *J* = 8.2 Hz)	115.56
A6	6.41 (dd, *J* = 8.2, 2.0 Hz)	120.83
A7	4.58 (d, *J* = 2.85 Hz)	46.41
A8	3.92 (d, *J* = 2.9 Hz)	48.32
A9		172.57
B3-OMe	3.80 s	56.39
B1		130.29
B2	6.80 s	112.95
B3		150.09
B4		146.20
B5	6.96 s	116.20
B6		124.17
B7	7.60 s	137.67
B8		125.62
B9		167.00

Compound **10**, HRMS: exact mass calculated for $[M + Na]^+$ ($C_{24}H_{26}O_8$) requires m/z 465.1525 and was found as m/z 465.1520.

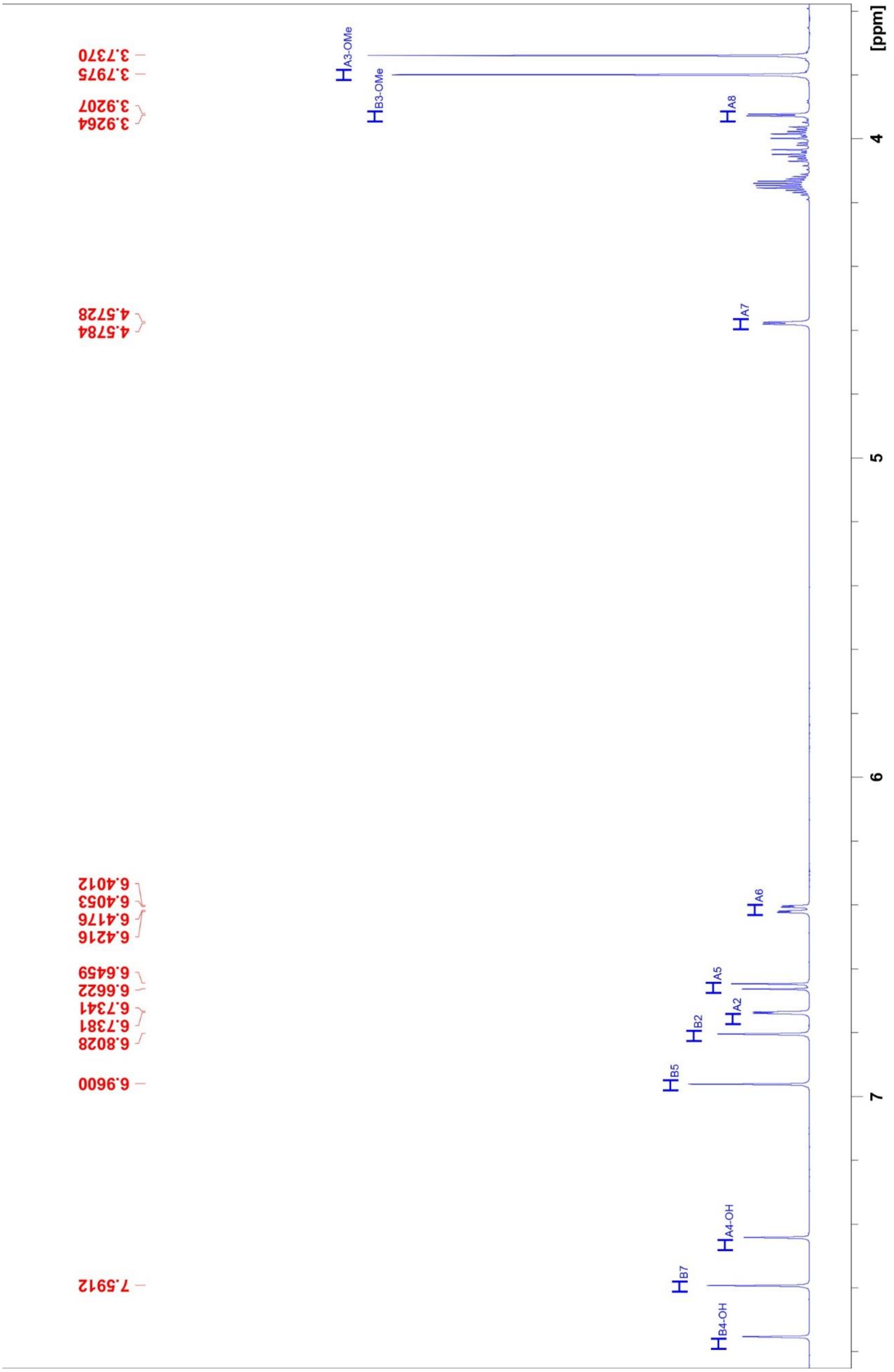

Figure S32. ^{1}H NMR spectrum of compound **10** in acetone.

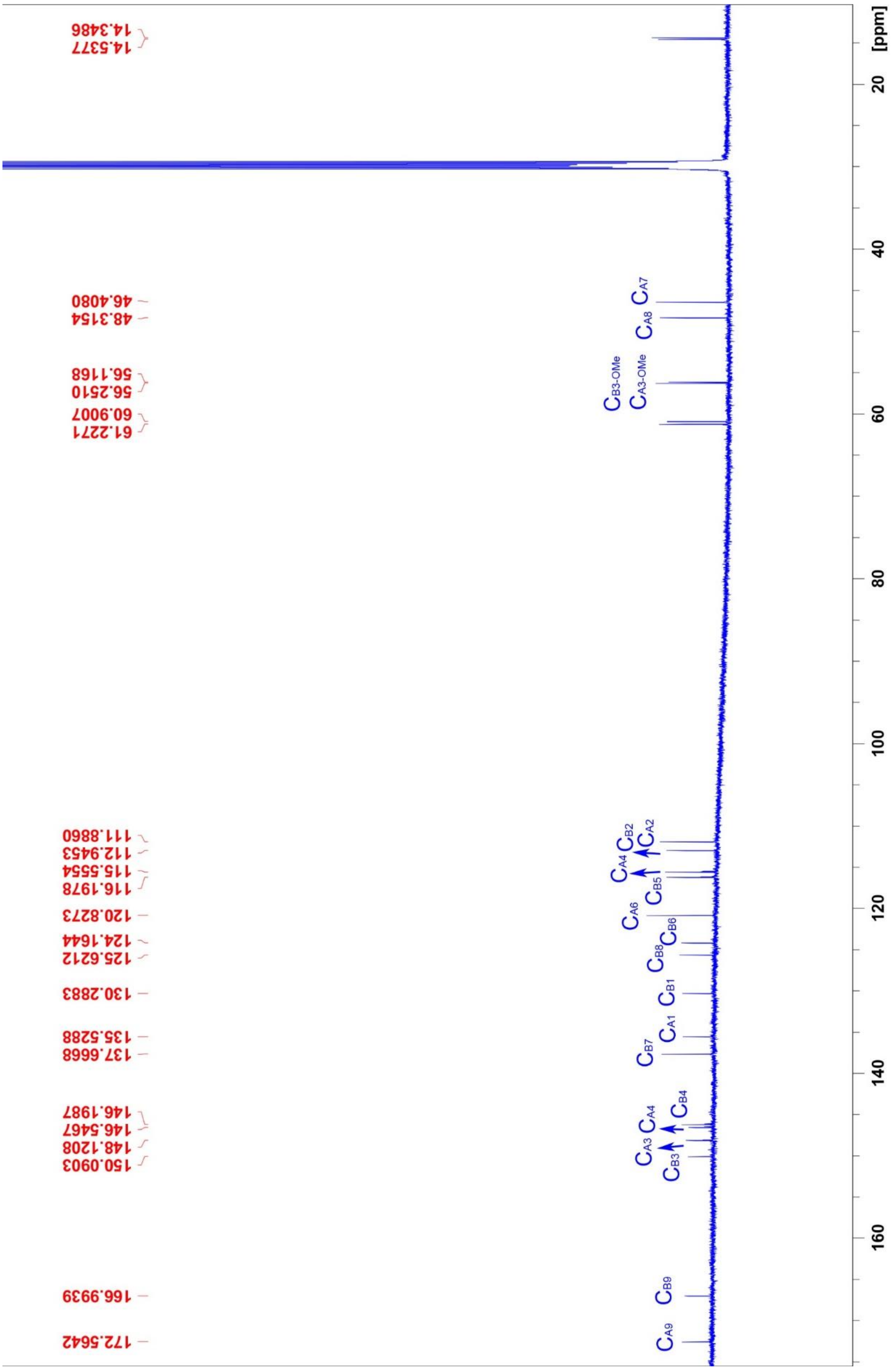

Figure S33. ^{13}C NMR spectrum of compound **10** in acetone.

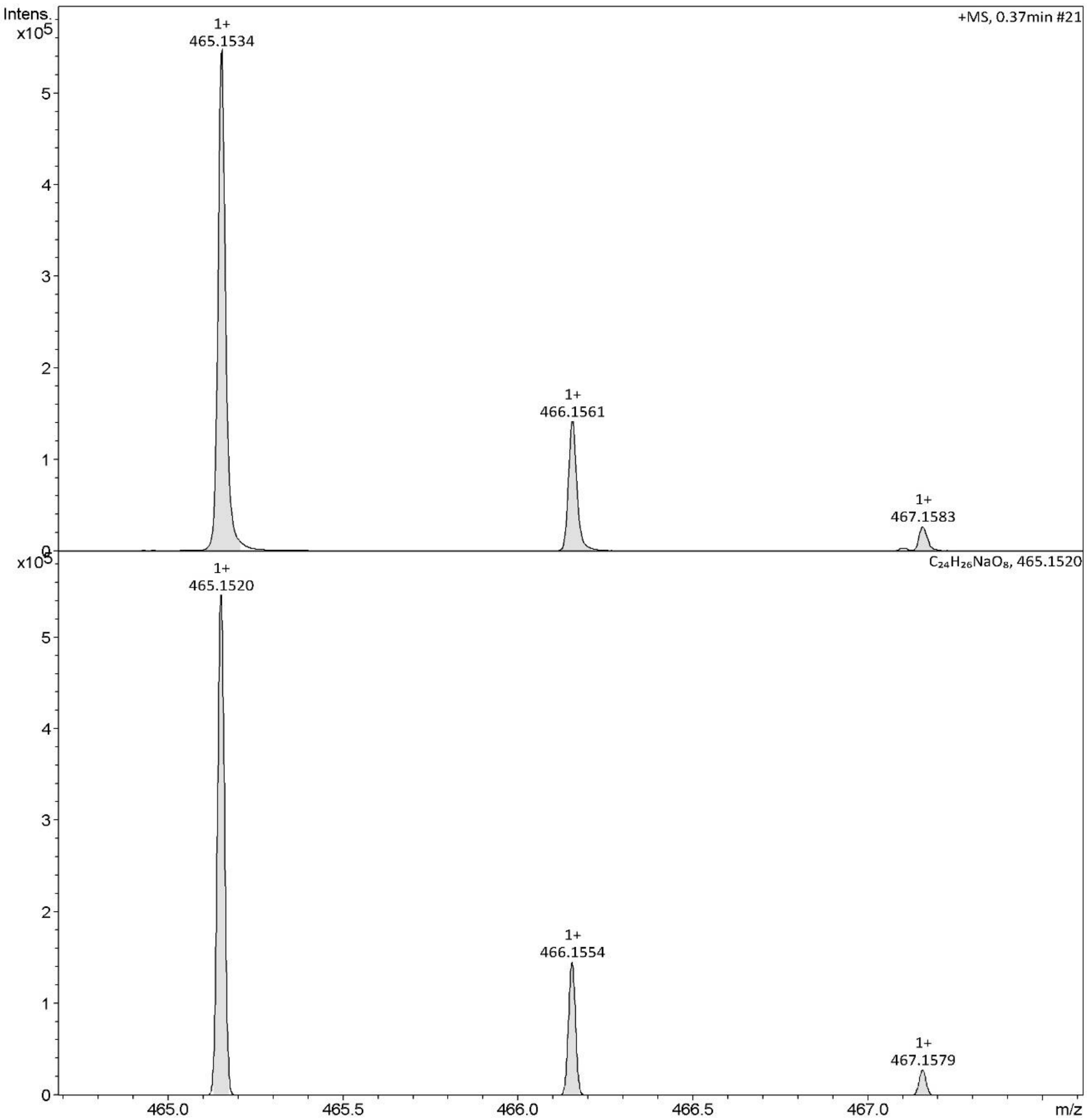

Figure S34. HRMS of compound **10**.

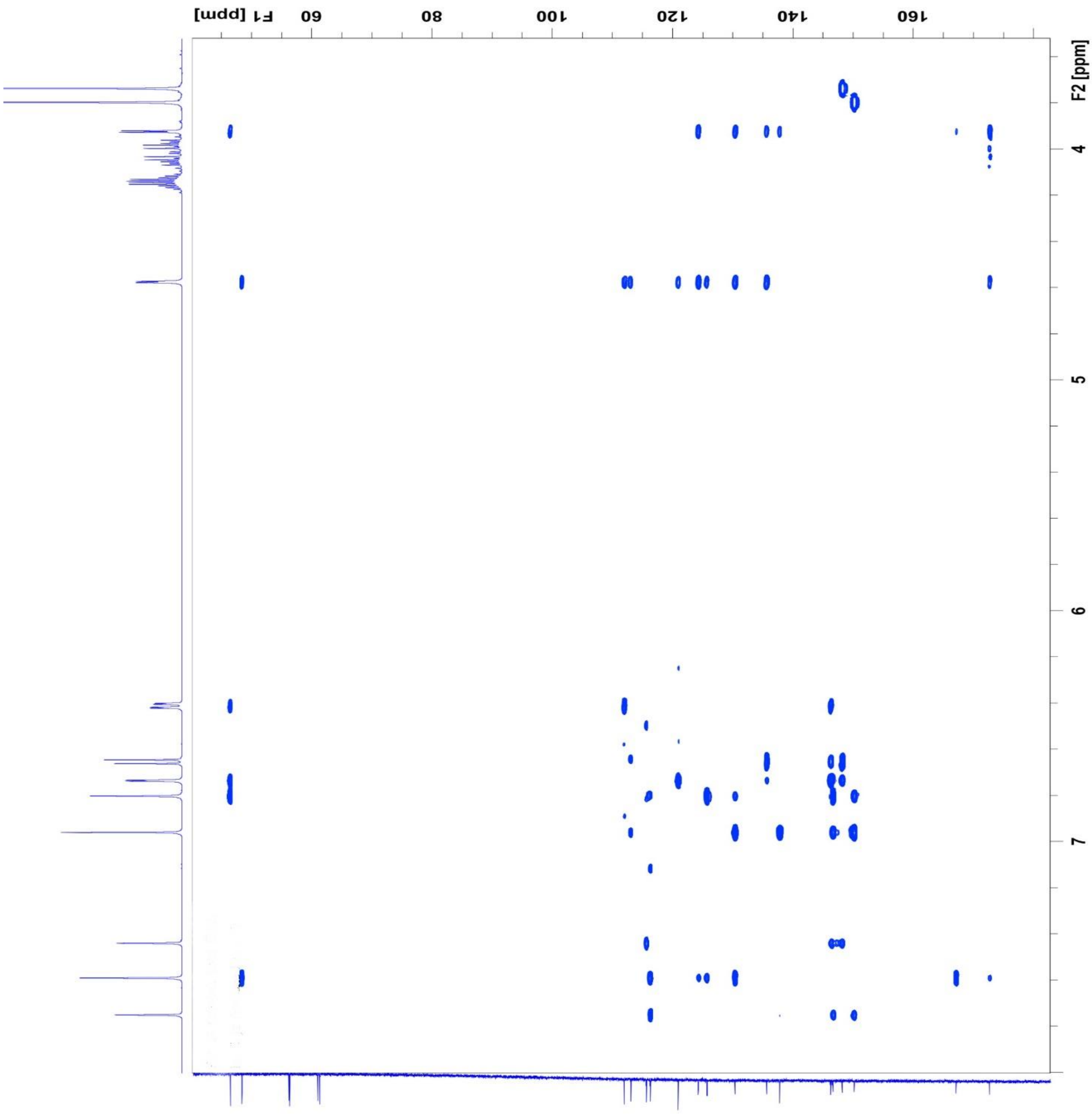

Figure S35. Two-dimensional HMBC spectrum of compound **10** in acetone.

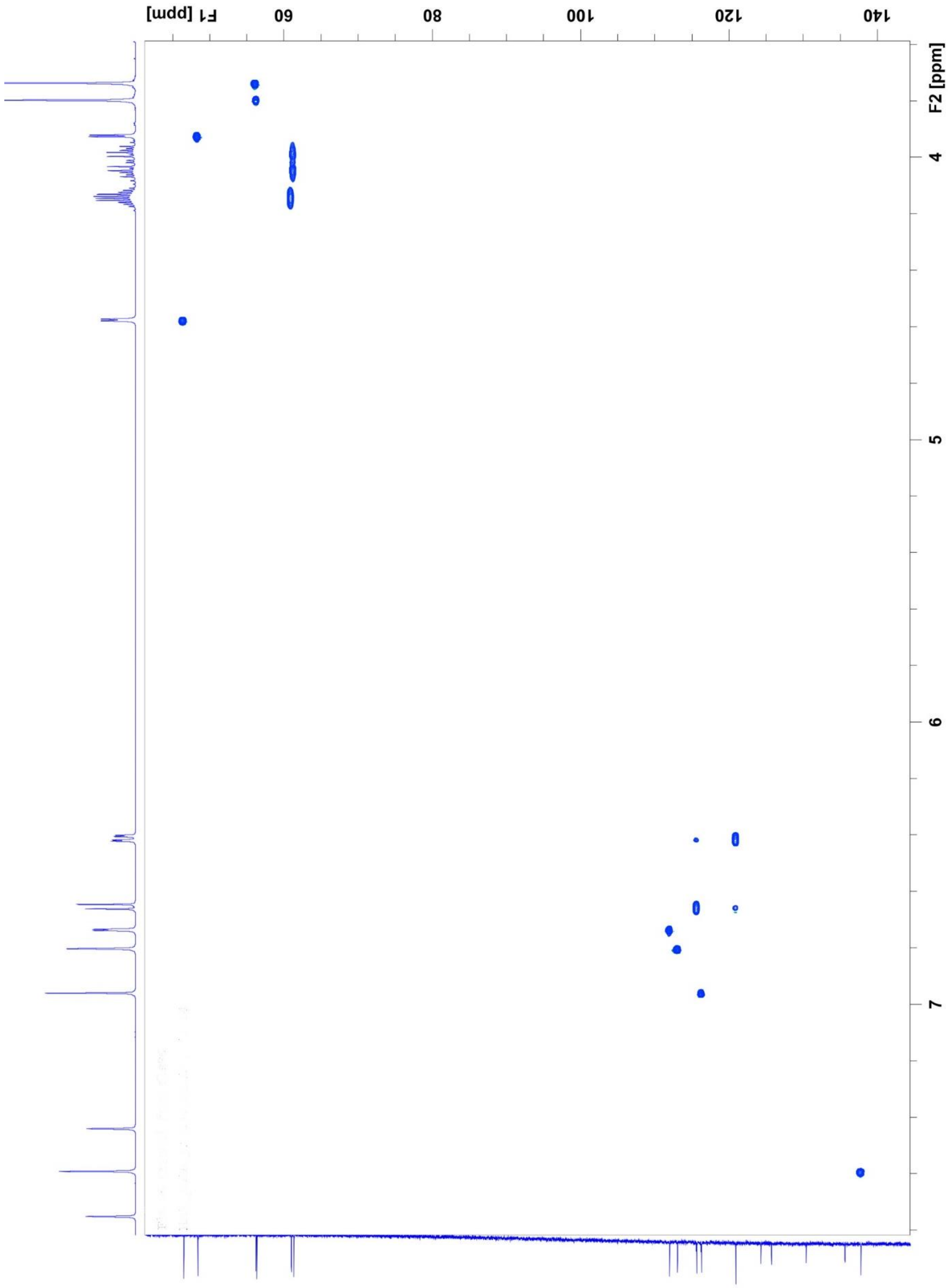

Figure S36. Two-dimensional HSQC spectrum of compound **10** in acetone.

References

1. Li, S.; Zhao, C.; Yue, F.; Lu, F. Revealing Structural Modifications of Lignin in Acidic γ-Valerolactone-H2O Pretreatment. *Polymers* **2020**, *12*, 116.
2. Ralph, J.; Quideau S.; Grabber J H.; Hatfield R. D. Identification and synthesis of new ferulic acid dehydrodimers present in grass cell walls. *J. Chem. Soc., Perkin Trans. 1* **1994**, *23*, 3485–3495.
3. Jia, Y.; He, Y.; Lu F. The structure-antioxidant activity relationship of dehydrodiferulates. *Food Chem.* **2018**, *269*, 480–485.
